# 'Off-the-shelf’ allogeneic antigen-specific adoptive T-cell therapy for the treatment of multiple EBV-associated malignancies

**DOI:** 10.1136/jitc-2020-001608

**Published:** 2021-02-15

**Authors:** Debottam Sinha, Sriganesh Srihari, Kirrliee Beckett, Laetitia Le Texier, Matthew Solomon, Archana Panikkar, George R Ambalathingal, Lea Lekieffre, Pauline Crooks, Sweera Rehan, Michelle A. Neller, Corey Smith, Rajiv Khanna

**Affiliations:** Immunology, QIMR Berghofer Medical Research Institute, Herston, Queensland, Australia

**Keywords:** immunity, cellular, immunotherapy, immunotherapy, adoptive, lymphocytes, tumor-infiltrating, t-lymphocytes

## Abstract

**Background:**

Epstein-Barr virus (EBV), an oncogenic human gammaherpesvirus, is associated with a wide range of human malignancies of epithelial and B-cell origin. Recent studies have demonstrated promising safety and clinical efficacy of allogeneic ‘off-the-shelf’ virus-specific T-cell therapies for post-transplant viral complications.

**Methods:**

Taking a clue from these studies, we developed a highly efficient EBV-specific T-cell expansion process using a replication-deficient AdE1-LMPpoly vector that specifically targets EBV-encoded nuclear antigen 1 (EBNA1) and latent membrane proteins 1 and 2 (LMP1 and LMP2), expressed in latency II malignancies.

**Results:**

These allogeneic EBV-specific T cells efficiently recognized human leukocyte antigen (HLA)-matched EBNA1-expressing and/or LMP1 and LMP2-expressing malignant cells and demonstrated therapeutic potential in a number of in vivo models, including EBV lymphomas that emerged spontaneously in humanized mice following EBV infection. Interestingly, we were able to override resistance to T-cell therapy in vivo using a ‘restriction-switching’ approach, through sequential infusion of two different allogeneic T-cell therapies restricted through different HLA alleles. Furthermore, we have shown that inhibition of the programmed cell death protein-1/programmed death-ligand 1 axis in combination with EBV-specific T-cell therapy significantly improved overall survival of tumor-bearing mice when compared with monotherapy.

**Conclusion:**

These findings suggest that restriction switching by sequential infusion of allogeneic T-cell therapies that target EBV through distinct HLA alleles may improve clinical response.

## Background

Epstein-Barr virus (EBV), a human B-lymphotropic oncogenic herpesvirus, is associated with multiple malignancies of both B-cell and epithelial cell origin, with an estimated 200,000 newly diagnosed cases annually (about 1.5% of all human cancer cases worldwide).^1^ These include Burkitt’s lymphoma, Hodgkin lymphoma (HL), natural killer or T (NK/T) cell lymphoma, post-transplant lymphoproliferative disease (LPD), nasopharyngeal carcinoma (NPC) and gastric carcinoma (GC).^2 3^ To date, radiation and/or chemotherapy remain the primary options for the treatment of EBV-associated malignancies. Of late, targeting viral antigens expressed in EBV-driven hematological malignancies using adoptive cellular therapy has demonstrated response rates of up to 80% in patients refractory to standard treatment.^4–6^ However, extension of this strategy to EBV-associated solid cancers of epithelial origin has achieved limited success.^7 8^ While the tumor microenvironment and disease burden are key factors impacting clinical outcomes,^9^ inefficiencies in autologous T-cell therapy manufacturing and targeting of malignant cells have emerged as major roadblocks for successful treatment of EBV-associated solid cancers.^10 11^ Many groups, including ours, have previously demonstrated that autologous T cells expanded with adenoviral vectors, synthetic peptide epitopes or EBV-transformed lymphoblastoid cell lines (LCLs) can be safely used for the treatment of EBV-associated malignancies.^12–16^ Using this approach in a phase I clinical study, we demonstrated disease stabilization in a majority of patients with NPC with stage IV refractory disease.^13^ In spite of these promising clinical results, we were unable to expand autologous T cells from a number of patients with NPC due to underlying immune deficiencies including severe lymphopenia.

To overcome the limitations of manufacturing autologous T-cell therapies, ‘off-the-shelf’ allogeneic virus-specific T cells expanded from healthy virus carriers have been proposed as an alternative therapeutic tool for the treatment of critically ill patients. Indeed, a number a groups including pioneering studies by Crawford and colleagues have successfully used these allogeneic virus-specific T cells to treat infectious complications in transplant recipients.^17–26^ More importantly, these T cells can be offered to patients rapidly, with minimal side effects. The successful translation of these findings to patients with solid cancers could have a major impact on the clinical management of patients whose tumors are resistant to standard therapies.^27 28^ Herein, we provide a preclinical assessment of an EBV-specific allogeneic T-cell therapy for multiple EBV-associated malignancies of different cellular origin, both in vitro and in vivo. We demonstrate that a ‘restriction-switching’ approach involving sequential infusion of two different allogeneic EBV-specific T-cell products, restricted through different human leukocyte antigen (HLA) types, can lead to better tumor control and overall survival. Furthermore, combination therapy based on blocking the programmed cell death protein-1 (PD-1)/programmed death-ligand 1 (PD-L1) axis and the administration of allogeneic EBV-specific T cells significantly improved tumor control and overall survival.

## Materials and methods

### Cell culture

The cell lines used in this study were cultured and maintained as per American Type Culture Collection (ATCC) recommendations, including incubation at 37°C with 20% O_2_ and 6.5% CO_2_. The NPC43 cell line was cultured in the presence of Rho-associated protein kinase (ROCK) inhibitor (Y-27632 at 4 µM), which enabled maintenance of the EBV copy number. When NPC43 cells are cultured in the absence of this inhibitor, the malignant cells lose EBV, as demonstrated by Lin *et al*.^29^ The HLA types of these cell lines are listed in table 1. All cell lines were regularly tested for mycoplasma infection and authenticated using short tandem repeat profiling by the Scientific Services Department at QIMR Berghofer Medical Research Institute.

**Table 1:** EBV-associated cancer cell lines used in the study

Cancer cell lines (origin)	HLA typing*	HLA-matched allogeneic EBV-specific T-cell products
SNU719 (GC)	A*24:02, 24:02; B*07:02, 52:01; C*07:02,12:02	TIG-001 and TIG-004
C17 (NPC)	A*02:01, 26:01; B*44:02,51:01; C*05:01,14:02	TIG-002
C666.1 (NPC)	B*58:02; C*03:04	TIG-003
SNT16 (NK/T)	A*02:01, 24:02; B*48:01, 52:01; C*08:03, 12:02	TIG-001 and TIG-002
YCLLE1 (GC)	A*24:02, 24:02; B*15:07, 40:01; C*03:03, 08:22	TIG-001 and TIG-002
GP202 (GC)	A*01:01, 24:02; B*08:01, 18:01; C*07:01, 07:01	TIG-001
NPC43 (NPC)	A*11:01, 11:01; B*50:01, 50:01; C*06:02, 06:02	TIG-006
L591 (HL)	A*01:01, 33:01; B*08:01, 35:03; C*03:04, 07:01	TIG-002
LCL-01 (B cells)	A*24:02, 24:02;B*08:02,50:01; C*05:02,07:02	TIG-001
LCL-02 (B cells) HEK293T	A*02:01,33:01; B*58:02,08:02; C*04:01,14:02 A*02:01,03:01; B07:02,35:01; C*07:02,07:02	TIG-002 and TIG-003 TIG-002

*HLA alleles matched between the cancer cell lines and allogeneic T-cell products are underlined.

EBV, Epstein-Barr virus; HLA, human leukocyte antigen; LCL, lymphoblastoid cell line.

### RNA extraction and quantitative real-time PCR

RNA was extracted and qRT-PCR was performed as reported previously.^30^ The primers comprised of LMP1: FP-5′CAGTCAGGCAAGCCTATGA3′, RP-5′CTGGTTCCGGTGGAGATGA-3′; LMP2: 5′-AGCTGTAACTGTGGTTTCCATGAC-3′, RP-5′-GCCCCCTGGCGA AGAG-3′; EBNA1: FP-5′-TACAGGACCTGGAAATGGCC-3′, RP-5′-TCTTTGAGGTCCACTGCCG-3′; HPRT1: FP-5′-CCTGGCGTCGTGATTAGTGAT-3′, RP-5'-AGACGTTCAGTCCTGTCCATAA-3'; 18sRNA: 5′-CGAAAGCATTTACCAAGGAC-3′, RP-5′-TTATTGTGTCTGGACCTGG-3′.

### Generation of T-cell bank

The latent membrane protein (LMP)/EBV-encoded nuclear antigen 1 (EBNA1)-specific allogeneic ‘off-the-shelf’ T-cell bank was generated as reported previously.^12 31^ Briefly, peripheral blood mononuclear cells (PBMCs) were harvested from 100 to 300 mL of venous blood from seropositive donors covering a wide HLA spectrum. The AdE1-LMPpoly vector was then used to infect 30% of the PBMC (MOI of 10:1) which were then irradiated and cocultured with the remaining PBMC for 2 weeks in a growth medium (RPMI 1640 medium supplemented with 10% fetal calf serum) supplemented with 120 IU/mL of recombinant interleukin 2 (IL-2, Komtur Pharmaceuticals, California, USA) every 3–4 days. On day 14 of cell culture, cells were harvested and cryopreserved. Before cryopreservation, cells were tested for sterility and antigen specificity. To analyze LMP1 and LMP2 and EBNA1 specificity, an intracellular cytokine assay was performed as reported previously.^12^ Phenotypic characterization was performed by surface staining the T-cell products using anti-CD3-APC (clone SK7, BD Biosciences, Victoria, Australia), anti-CD4-FITC (clone RPA-T4, BD Biosciences), anti-CD8-PerCP-Cy5.5 (clone RPA-T8, eBioscience, California, USA), anti-CD19-APC-Cy7 (clone HIB19, BioLegend) and anti-CD56-BV421 (clone SNCAM16.2, BD Biosciences). In addition, these T cells were also assessed for alloreactivity using K562 cells expressing HLA class I alleles (see below). Flow cytometry was performed using the BD LSRFortessa with FACSDiva software (BD Biosciences) and analyzed using FlowJo software (TreeStar, California, USA).

### T-cell alloreactivity assay

Each of the off-the-shelf EBV-specific T-cell therapy products was assessed for any potential alloreactivity using K562 cells expressing individual HLA class I alleles. Briefly, 1×10^6^ K562 cells were electroporated with 1 µg of pEGFP-N1 plasmid DNA encoding HLA class I allele(s) using Amaxa Cell line Nucleofector Kit (Lonza Bioscience, Victoria, Australia). These K562 cells were cultured in growth medium (RPMI with 10% FCS) containing G418 (600 µg/mL) for 2–3 weeks and then assessed for stable HLA transgene expression by analyzing green fluorescent protein expression using flow cytometry. For alloreactivity evaluation, the T-cell therapy products were incubated with individual K562 HLA class I transfectants (effector to target ratio: 10:1) for 4 hours in the presence of GolgiPlug (BD Biosciences) and were assessed for the intracellular production of interferon-γ (IFN-γ) using flow cytometry. Cell staining was performed using anti-CD3-APC, anti-CD4-FITC, anti-CD8-PerCP-Cy5.5 (described above) and anti-IFN-γ-AF700 (clone B27, BD Biosciences).

### Cell viability assay

A cell viability assay was performed using the CellTiter 96 Aqueous One Solution Reagent (Promega, Victoria, Australia) with three biological replicates per EBV-associated cancer cell line, in triplicate.^32^ Briefly, the cancer cells (target cells) were plated at a density of 5000 cells/well in an overall media volume of 200 µL in a 96-well tissue culture plate (BD Biosciences). The AdE1-LMPpoly-transfected effector T cells were freshly thawed and resuspended in RPMI-1640 with 10% FCS and 120 IU/mL of recombinant IL-2. T cells were incubated for 24 hours at 37°C and 6.5% CO_2_, prior to combining them with target cells at effector-to-target ratios between 5:1 and 100:1. The Aqueous One Solution Reagent was added to each well (1:100 dilution in media) and the plate was incubated for 1 hour prior to assessing the optical density at 490 nm using a microplate reader.^33^

### Cytotoxicity assay

Cytotoxicity assays were performed using the CytoTox 96 Non-Radioactive Cytotoxicity Assay Kit (Promega) according to the manufacturer’s instructions. Assays included three biological replicates per EBV-associated cancer cell line, in triplicate.^34^ Briefly, the cancer cells (target cells) were plated at a density of 5000 cells/well in an overall media volume of 200 μL in a 96-well tissue culture plate, using similar conditions to the cell viability assay. Following termination with stop solution, the absorbance of the mixture at an optical density of 490 nm was measured via a microplate reader (Bio-Rad, California, USA).

### Polychromatic phenotypic profiling of AdE1-LMPpoly-generated T-cell products and cancer cells

EBV-associated cancer cells were plated at a density of 1×10^5^ cells/well. After 24 hours, T cells were added at an effector-to-target ratio of 50:1 and the culture was incubated for 24 hours at 37°C and 6.5% CO_2_. To assess the impact of T cells on cancer cells, the cultured cells were then incubated at 4°C with the following antibodies: human anti-CD45-V500 (clone HI30, BD Biosciences), anti-CD3-AF700 (clone HIT3a, BioLegend), anti-CD56-BV421, anti-CD8-PerCP-Cy5.5, anti-CD19-APC-Cy7, anti-perforin-PE (clone dG9, eBioscience), anti-granzyme K-FITC (clone G3H69, BD Biosciences), anti-granzyme B-BV711 (clone GB11, BD Biosciences), Ki67-BV421 (clone B56, BD Biosciences), anti-active caspase-3-BV605 (clone C92-605, BD Biosciences) and LIVE/DEAD Fixable Near-IR Dead Cell Stain (Thermo Fisher Scientific, MA). Flow cytometry was performed using a BD LSRFortessa with FACSDiva software and postacquisition analysis was performed using FlowJo software.

### Animal housing

All animal work was approved by the QIMR Berghofer Medical Research Institute Animal Ethics Committee (number A0707-606M) and was performed in strict accordance with the Australian Code for the Care and Use of Animals for Scientific Purposes. All experimental animals were housed at the QIMR Berghofer Medical Research Institute Animal Facility in OptiMICE caging (Centennial, Colorado, USA) on a 12 hours light–dark cycle at 25°C. Dried granule food was sterilized by irradiation. The mice had free access to food and sterile water.

### In vivo assessment of the therapeutic efficacy of allogeneic EBV-specific T cells

In this study, 7–8-week-old female nonobese diabetic/severe combined immunodeficient (NOD-SCID) mice were subjected to irradiation with 0.3 Gy cobalt-60 and after 4 hours, were subcutaneously injected with 5×10^6^ EBV-associated cancer cells. The mice were monitored for tumor growth, weight and body condition score three times per week. Once the tumor was palpable, the mice were randomized into groups and were treated with 2×10^7^ tumor HLA-matched allogeneic EBV-specific T cells. The tumor size in these mice was measured three times per week using vernier calipers. To calculate tumor area, the formula B×S was used, where B=largest tumor measurement and S=smallest tumor measurement, based on two‐dimensional caliper measurements as previously described.^32^

### In vivo assessment of the therapeutic efficacy of allogeneic EBV-specific T cells in a humanized mouse model

Human cord blood was obtained from the placentas of full-term newborns after written parental consent, with ethical approval from the human research ethics committees of Mater Misericordiae Ltd and QIMR Berghofer. CD34^+^ cells were enriched using immunomagnetic beads according to the manufacturer’s instructions (CD34-positive selection kit, Miltenyi Biotec, Bergisch-Gladbach, Germany). Female NOD-Rag1^null^ IL2rg^null^ (NRG) mice of 7–8-weeks old were irradiated twice with 275 cGy, 3–4 hours apart, following which they were intravenously injected with 5×10^4^ CD34^+^ cells (HLA matched to the AdE1-LMPpoly-generated T cells used for treatment) per mouse with a 29-gauge needle. The mice were monitored twice weekly for body weight, body condition score and adverse reactions including graft-versus-host disease. In addition, tail vein bleeds were performed at weeks 4, 8, 10 and 12 to monitor the reconstitution of the human immune system. To assess immune reconstitution, cell surface phenotyping was performed using human anti-CD45-V500, mouse anti-CD45-V450 (clone 30-F11), anti-CD3-APC, anti-CD4-AF700 (clone RPA-T4), anti-CD8-PerCP-Cy5.5, anti-CD14-FITC (clone MфP9), anti-CD19-Pe-Cy5 (clone HB19), anti-CD23-BV786 (clone M-L233), and anti-CD56-BV650. These antibodies were supplied by BD Biosciences. HLA-A24 and HLA-B7 staining was performed using anti-HLA Bw4 (REA274: Meltenyi Biotec, Macquarie Park NSW, Australia) and anti-HLA Bw6 (HB-165;SFR8-B6, ATCC, Manassas, Virginia) specific antibodies followed by phycoerythrin(PE)-labeled goat anti-mouse IgG (Biolegend PE Cat#405307) or PE-labeled anti-rat IgM (BD Biosciences; Clone: G53-238), respectively. At the 12th week of reconstitution, the humanized NRG mice were intravenously injected with EBV (B95.8 strain) at 2.3×10^5^/mL median tissue culture infectious dose in 100 µL phosphate-buffered saline (PBS) under non-anesthetic conditions using a 29-gauge needle. On the 13th day post EBV infection, the mice were treated with 2×10^7^ HLA-matched AdE1-LMPpoly-generated T cells. These mice were monitored for 14 days post T-cell treatment, following which the mice were culled and their spleens were analyzed for tumor burden by determining the viral load as previously described.^35^

### Immunohistochemistry

For histological examination, tissues were collected and fixed in 4% formaldehyde (Sigma Aldrich, Missouri, USA) and immunohistology was performed as reported previously.^30^ The slides were scanned on the Aperio Scanscope XT, with a 40× objective. The CD3 staining was performed using unconjugated mouse anti-human CD3 antibody (clone F7.2.38, Agilent Dako, California, USA).

### Gene signature profile using NanoString and infiltrate profiling

A total of six 8-week-old female NOD-SCID mice were subcutaneously injected with EBV-positive cancer cell lines as described above. Once the tumor size reached 40 mm^2^, the mice were treated with 2×10^7^ EBV-specific T cells. After 5 days, tumors were harvested and labeled with human anti-CD45-V500, anti-CD3-APC, anti-CD4-PE and anti-CD8-PerCP-Cy5.5, and mouse anti-CD45-V450. Tumor-infiltrating CD8^+^ T cells were sorted using a FACSAria III (BD Biosciences) and RNA was extracted from sorted cells (RNeasy Mini Kit, QIAGEN, Victoria, Australia). Gene expression analysis was performed using a customized NanoString immune gene expression panel (NanoString Technologies, New South Wales, Australia). For each sample, 50 ng of total RNA in a final volume of 5 µL was mixed with a 3′ biotinylated capture probe alongside a 5′ reporter probe tagged with a fluorescent barcode from the custom gene expression code set. Probes and target transcripts were hybridized at 65°C for 12–16 hours. Hybridized samples were run on the NanoString nCounter Prep Station (NanoString Technologies) using the manufacturer’s recommended protocol, in which excess capture and reporter probes were removed and transcript-specific ternary complexes were immobilized on a streptavidin-coated cartridge. The samples were scanned at maximum scan resolution on the nCounter Digital Analyzer (NanoString Technologies). Data were processed using nSolver Analysis Software and the nCounter Advanced Analysis module (NanoString Technologies). For gene expression analysis, data were normalized using the geometric mean of housekeeping genes selected by the GeNorm algorithm (NanoString Technologies).

### Statistical analysis

The Student’s t-test and one-way analysis of variance with Bonferroni post hoc or Mann-Whitney U test (specified in figure legend) were performed using Prism V.6.0 software (GraphPad, California, USA), with p values calculated as indicated in figure legends.

## Results

### In vitro recognition of EBV-positive cancers by allogeneic ‘off-the-shelf’ virus-specific T cells

**Figure 1:**
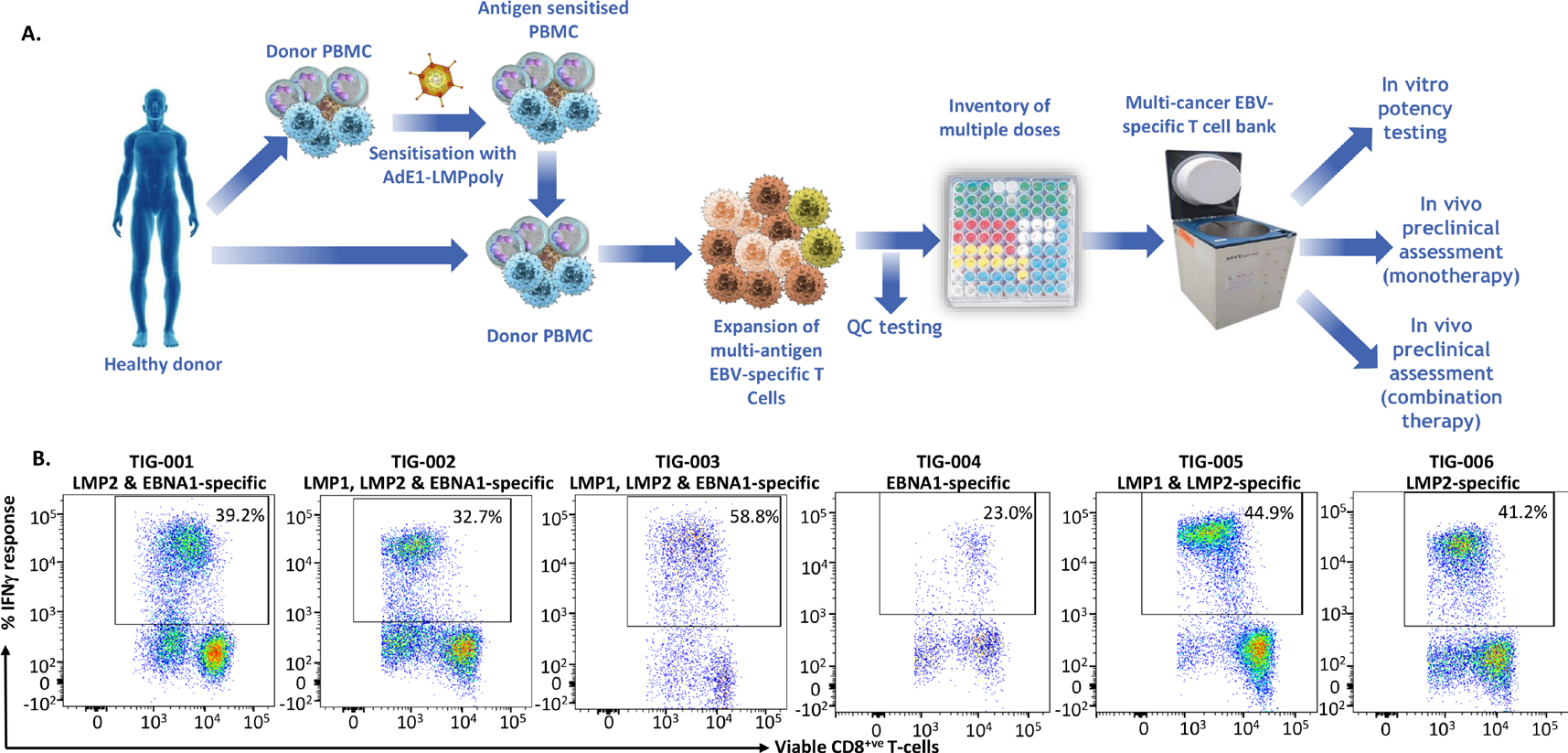
Generation of allogeneic ‘off-the-shelf’ EBV-specific T cells using AdE1-LMPoly. (A) Schematic representing the process for manufacturing the ‘off-the shelf’ allogeneic EBV-specific T-cell bank (TIG-001–006) using PBMC isolated from seropositive healthy donors. AdE1-LMPpoly vector was used to infect 30% of the PBMC (MOI of 10:1), which were then irradiated and cocultured with the remaining PBMC for 2 weeks. These T-cell cultures were supplemented every 2–3 days with growth medium containing recombinant IL-2. On the 14th day, T cells were cryopreserved and assessed for EBV-specific reactivity. (B) T cells were stimulated with a peptide pool containing EBNA1, LMP1 and LMP2 peptide epitopes and then assessed for intracellular IFN-γ expression. Representative flow cytometry plots show the percentage of CD8^+^ T cells demonstrating EBV epitope-specific reactivity in TIG-001-006. EBNA1, EBV-encoded nuclear antigen 1; EBV, Epstein-Barr virus; IFN-γ, interferon-γ; IL-2, interleukin 2; LMP1, latent membrane protein 1; LMP2, latent membrane protein 1; PBMC, peripheral blood mononuclear cell.

To assess immune recognition of HLA-matched EBV-positive cancer cells by allogeneic antigen-specific T cells, we generated six EBV-specific T-cell products using an adenoviral vector encoding truncated EBNA1 protein and multiple LMP1 and LMP2 epitopes as a polyepitope (referred to as AdE1-LMPoly)^12 13 36^ as outlined in figure 1A. These allogeneic AdE1-LMPoly-expanded T-cell products (TIG-001-006) were comprised of a median of 97.06% CD3^+^ T cells, of which 32.28% (median) were CD8^+^ T cells while 64.92% (median) were CD4^+^ T cells (online supplemental figure 1). The remaining population was mainly comprised of NK cells (online supplemental figure 1). These T-cell products were assessed for EBV antigen specificity using HLA-restricted pooled peptide epitopes from each antigen (LMP1, LMP2 and EBNA1). Data presented in figure 1B show EBV antigen-specific reactivity of each T-cell product. The antigen specificities and HLA restrictions of the T-cell products are shown in table 2 and their HLA typing is shown in online supplemental table 1. The products were also assessed for alloreactiviy against a panel of K562 cells expressing individual HLA class I alleles. Data presented in online supplemental figure 2 show that none of the T-cell products were alloreactive.

10.1136/jitc-2020-001608.supp1 Supplementary data

10.1136/jitc-2020-001608.supp2 Supplementary data

**Table 2:** EBV antigen specificities and HLA restrictions of allogeneic T cells used in the study

Allogeneic EBV-specific T-cell product	EBV antigen specificity (HLA restriction)
TIG-001	LMP2 (SSCSSCPLSK/A*11:01, TYGPVFMCL/A*24:02) and EBNA1 (FVYGGSKTSL/C*03:04)
TIG-002	LMP1 (YLLEMLWRL/A*02:01, YLQQNWWTL/A*02:01); LMP2 (FLYALALLL/A*02:01, IEDPPFNSL/B*40:01); EBNA1 (FVYGGSKTSL/C*03:04)
TIG-003	LMP1 (IALYLQQNW/B*58:01); LMP2 (IEDPPFNSL/B*40:01, MSNTLLSAW/B*58:01); EBNA1 (FVYGGSKTSL/C*03:04)
TIG-004	EBNA1 (RPQKRPSCIGC /B*07:02)
TIG-005	LMP1 (YLLEMLWRL/A*02:01); LMP2 (FLYALALLL/A*02:01, PYLFWLAAI/A*23:01)
TIG-006	LMP2 (SSCSSCPLSK/A*11:01)

EBNA1, EBV-encoded nuclear antigen 1; EBV, Epstein-Barr virus; HLA, human leukocyte antigen; LMP1, latent membrane protein 1; LMP2, latent membrane protein 2.

**Figure 2:**
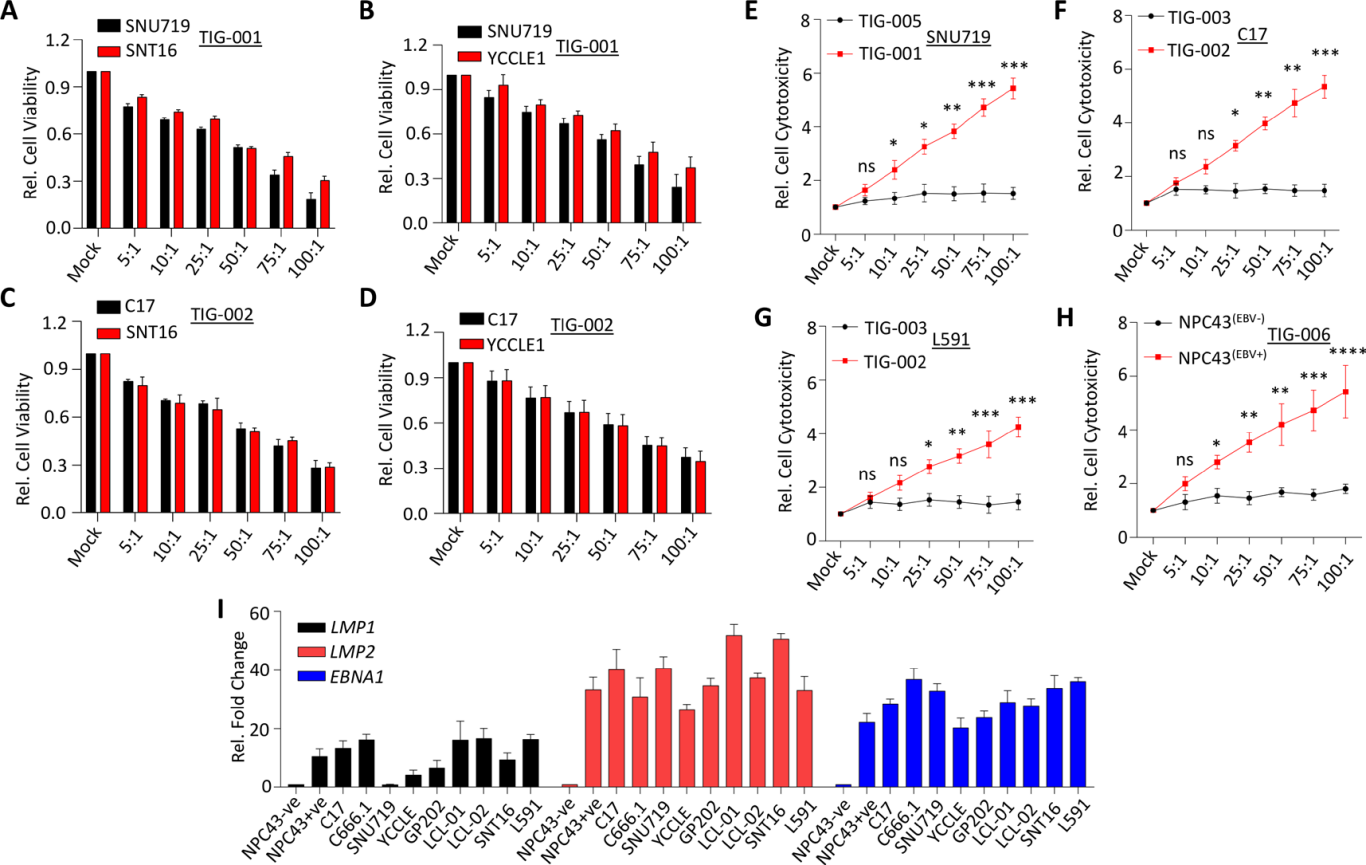
In vitro recognition of multiple EBV-associated cancer cells by HLA-matched allogeneic EBV-specific T-cells. (A–D) Cell viability was measured by MTS assay following the exposure for 24 hours of EBV-positive cancer cells SNU719, SNT16 (A), SNU719 and YCCLE1 (B), C17 and SNT16 (C) and C17 and YCCLE1 (D) to HLA-matched TIG-001 and TIG-002 allogeneic EBV-specific T cells across varying effector-to-target (E:T) ratios (5:1–100:1). (E–H) Cytotoxicity was measured by LDH release following the exposure for 24 hours of EBV-positive and EBV-negative cancer cells SNU719 (E), C17 (F), L591 (G) and EBV-positive and EBV-negative NPC43 cells (H) to HLA-matched and HLA-mismatched allogeneic EBV-specific T cells across varying E:T ratios (5:1–100:1). HLA alleles that were matched between the T-cell products and the cancer cell lines are underlined in table 1 and online supplemental table 1. (I) Representation of relative fold expression of *LMP1*, *LMP2* and *EBNA1* at mRNA level in each cell line. The expression of these genes is represented as a relative fold change in EBV-positive with respect to EBV-negative (NPC43^–^ cancer cells). Housekeeping genes *HPRT1* and *18sRNA* were used as controls. PBS was used as mock treatment across all experiments. Error bars corresponds to mean ± SD from three independent experiments. P values were calculated using Student’s t-test. *p<0.05, **p<0.01, ***p<0.001. EBNA1, EBV-encoded nuclear antigen 1; EBV, Epstein-Barr virus; HLA, human leukocyte antigen; LDH, lactate dehydrogenase; LMP1, latent membrane protein 1; LMP2, latent membrane protein 2; NPC, nasopharyngeal carcinoma; ns, not significant.

Each of these EBV-specific T-cell products was tested against a panel of EBV-associated cancers (table 1) including NPC, NK/T-cell lymphoma, GC, HL and EBV-transformed LCLs using lactate dehydrogenase cytotoxicity and tetrazolium-based cell proliferation (3-(4,5-dimethylthiazol-2-yl)-5-(3-carboxymethoxyphenyl)-2-(4-sulfophenyl)-2H-tetrazolium (MTS)) assays. Data presented in figure 2A–H and online supplemental figure 3A–H show that the allogeneic EBV-specific T cells efficiently recognized HLA-matched EBV-positive cancer cells of both epithelial and lymphoid origin. More importantly, each of the T-cell products recognized multiple HLA-matched cancer cells and this immune recognition was evident in both cytotoxicity and cell proliferation assays (figure 2A–H; online supplemental figure 3A–H). The specificity of immune recognition by the allogeneic EBV-specific T cells was confirmed by their selective recognition of EBV-positive NPC43 cancer cells, while EBV-negative NPC43 cancer cells were not recognized (figure 2H). These observations were consistent with the EBV gene expression in the malignant cells (figure 2I). In addition, we observed no cytotoxicity when EBV-negative HEK293T cells were exposed to HLA-matched and HLA-mismatched allogeneic EBV-specific T cells in a dose-dependent manner (online supplemental figure 3I). Furthermore, we did not observe any significant difference in cell viability of EBV-positive tumor cells (C17 and YCCLE1) when exposed to HLA-matched cytomegalovirus-specific T cells in a dose-dependent manner (online supplemental figure 3J).

**Figure 3:**
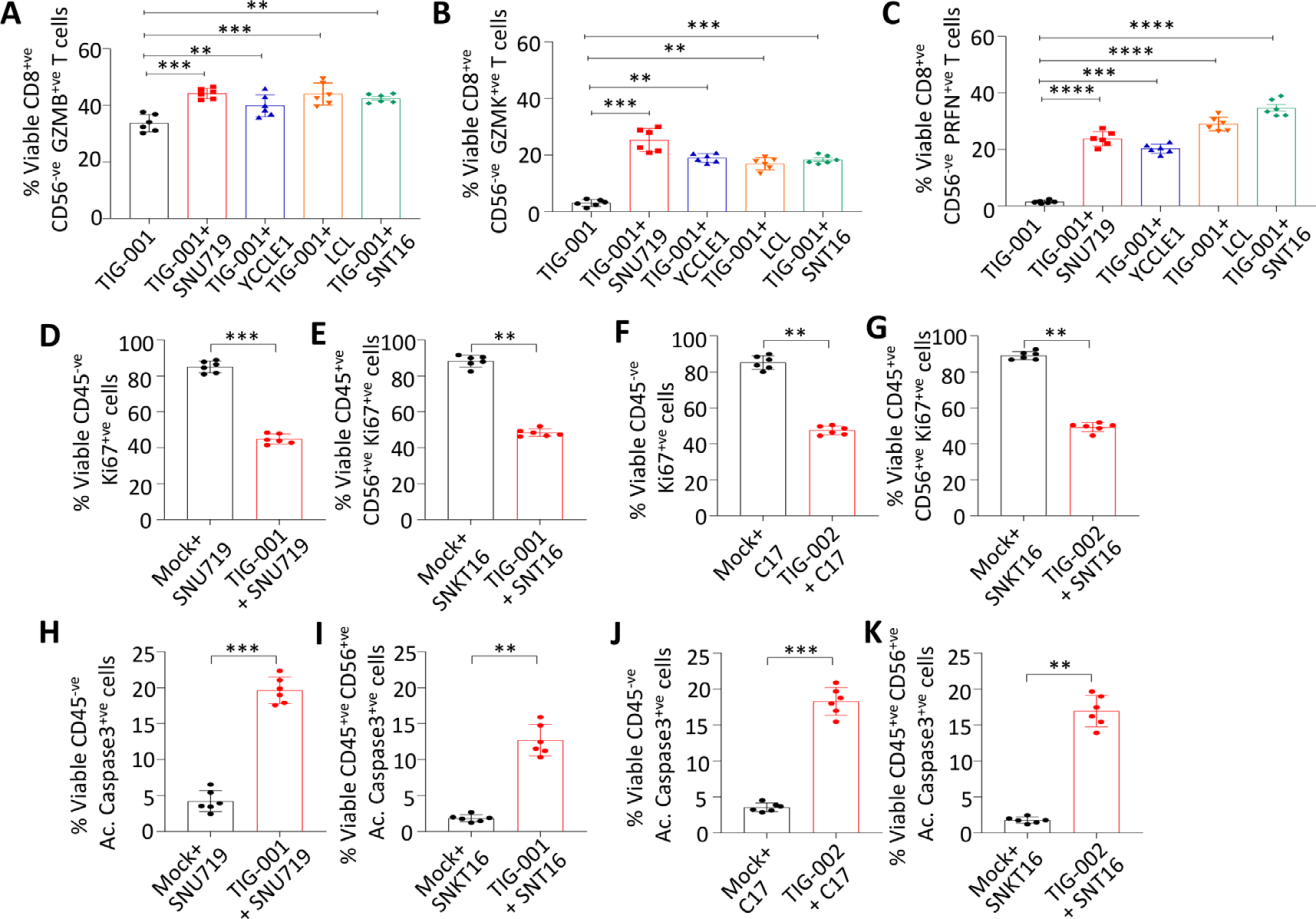
Impact of immune interaction between allogeneic EBV-specific T cells and HLA-matched EBV-positive malignant cells. (A–C) The expression of effector molecules including granzyme B, granzyme K and perforin in allogeneic EBV-specific T cells following exposure for 24 hours to HLA-matched EBV-positive cancer cells (E:T of 50:1). Error bars represent the mean ± SD from three independent experiments. P values were calculated using two-way ANOVA. (D–G) The proliferation of SNU719 (D), SNT16 (E), C17 (F) and SNK16 (G) cells following exposure to EBV-specific T cells (TIG-001 or TIG-002), based on Ki67 expression. (H–K) The assessment of active caspase-3^+^ cells within SNU719 (H), SNT16 (I), C17 (J) and SNT16 (K) cells following exposure to EBV-specific T cells (TIG-001 or TIG-002). PBS was used as mock treatment across all experiments. HLA alleles that were matched between the T-cell products and the cancer cell lines are underlined in table 1 and online supplemental table 1. Error bars represent the mean ± SD from three independent experiments. P values were calculated using Mann-Whitney test: **p<0.01; ***p<0.001; ****p<0.0001. ANOVA, analysis of variance; EBV, Epstein-Barr virus; HLA, human leukocyte antigen.

In the next set of experiments, we characterized the impact of immune interaction between allogeneic EBV-specific T cells and HLA-matched EBV-positive malignant cells on the expression of effector molecules, cellular proliferation and the cell death-associated maker caspase-3. For this analysis, we employed a gating strategy (online supplemental figure 4A–C) which selectively allowed us to analyze the expression of multiple markers on cancer cells and T cells. Data presented in figure 3A–C and online supplemental figure 4D–F show that allogeneic EBV-specific T cells upregulated expression of multiple effector molecules including granzyme B (GzmB), granzyme K (GzmK) and perforin (Prf) following exposure to HLA-matched EBV-positive cancer cells. Simultaneously, we also observed a significant reduction in the cancer cell proliferation rate, as indicated by a reduction in the number of Ki67-positive cancer cells (figure 3D–G), and a significant increase in active caspase-3 staining, suggesting that EBV-specific T cells caused increased tumor cell death (figure 3H–K). In addition, no significant difference in expression of GzmB, GzmK and Prf was observed when EBV-negative HEK293T cells were exposed to HLA-matched allogeneic EBV-specific T cells (online supplemental figure 4G-I). Taken together, these results demonstrate that in vitro-expanded allogeneic EBV-specific T cells can efficiently recognize EBV-positive cancer cells regardless of their cellular origin.

### Assessment of therapeutic efficacy of allogeneic EBV-specific cytotoxic T cells in vivo

**Figure 4:**
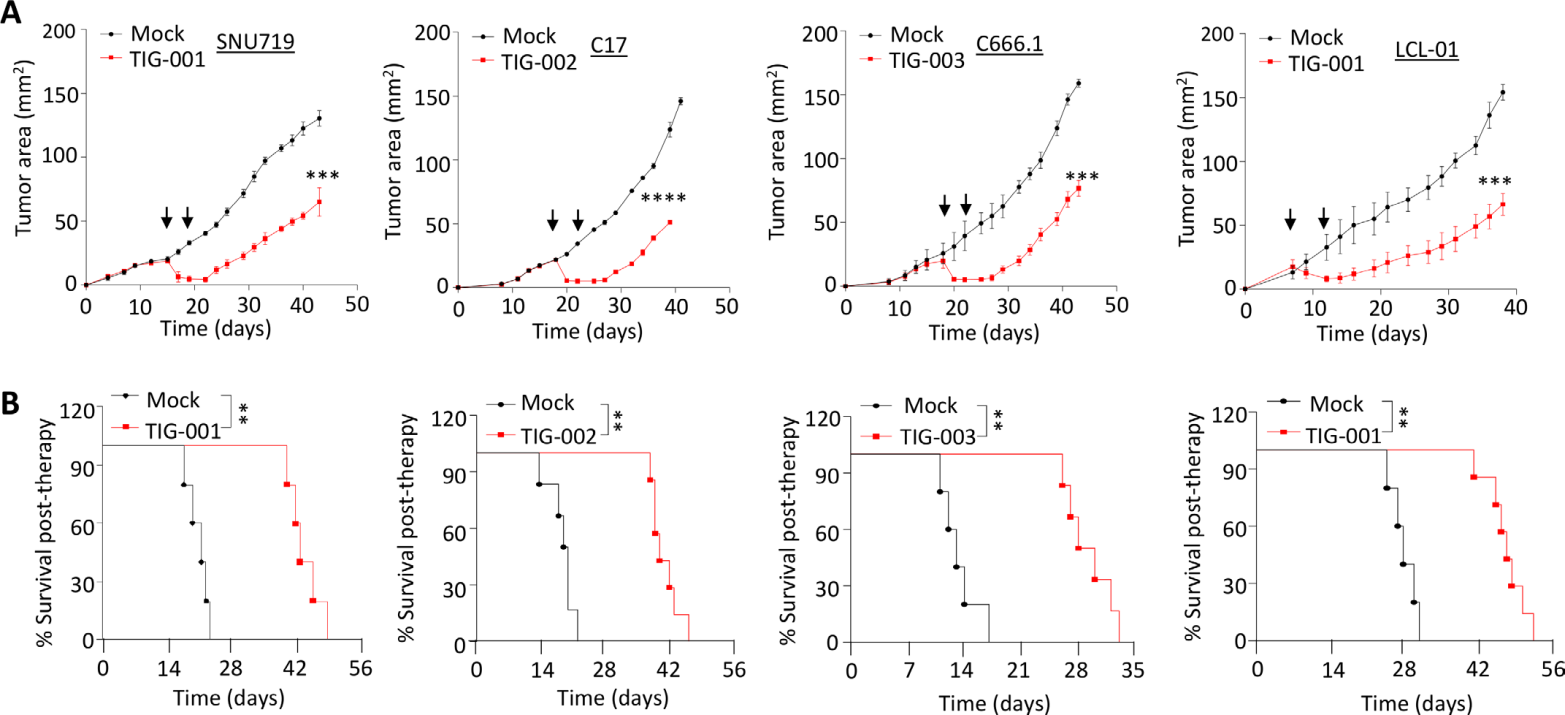
Assessment of therapeutic efficacy of allogeneic EBV-specific cytotoxic T cells in vivo. (A) The impact of adoptive T-cell therapy using allogeneic EBV-specific T cells on the outgrowth of EBV-positive SNU719 (n=8), C17 (n=9), C666.1 (n=9) and LCL (n=9) tumors in NOD-SCID mice was assessed. Tumor-bearing mice were treated with two doses of HLA-matched T cells (2×10^7^ T cells/dose/mouse). The growth of each xenograft is represented as the mean tumor area ± SD. (B) Kaplan-Meier overall survival analysis of the mice bearing EBV-associated tumors as described in (A) following adoptive T-cell therapy. PBS was used as mock treatment across all experiments. HLA alleles that were matched between the T-cell products and the cancer cell lines are underlined in table 1 and online supplemental table 2. The animals’ survival (n≥4 mice/group) was monitored over the indicated period of time and statistical significance was analyzed by log‐rank test: **p<0.01; ***p<0.001;****p<0.0001. EBV, Epstein-Barr virus; HLA, human leukocyte antigen.

Having demonstrated efficient recognition of multiple EBV-associated cancers by allogeneic EBV-specific T cells in vitro, we next assessed the therapeutic potential of these effector cells in vivo. Using xenograft models for GC (SNU719), NPC (C17 and C666.1) and lymphoma (LCL) in immunodeficient NOD-SCID mice, we first adoptively transferred HLA-matched allogeneic EBV-specific T cells (2×10^7^ T cells for each infusion/animal) when tumors in each mouse reached 25 mm^2^. These animals were monitored for tumor outgrowth until tumors reached 150 mm^2^ in mock-treated mice (infused with PBS alone). We observed that the animals treated with allogeneic EBV-specific T cells demonstrated a significant reduction in tumor outgrowth when compared with mock-treated mice, which was uniform across all three models (figure 4A). This reduction in tumor outgrowth was coincident with the infiltration of adoptively transferred T cells as indicated by the presence of CD3^+^ T cells in tumor sections from the three models (online supplemental figure 5). More importantly, mice treated with allogeneic EBV-specific T cells also showed a significant increase in overall survival when compared with mock-treated mice (figure 4B). Furthermore, we did not observe any adverse effects of allogeneic EBV-specific T-cell therapy as indicated by the animals’ body weight and the weight of their liver, spleen and kidney when compared with mice treated with PBS alone (online supplemental figure 6). These observations highlight the therapeutic efficacy and safety of allogeneic EBV-specific T cells in multiple tumor models.

### Improved efficacy of allogeneic EBV-specific T cells following ‘switch’ therapy

**Figure 5:**
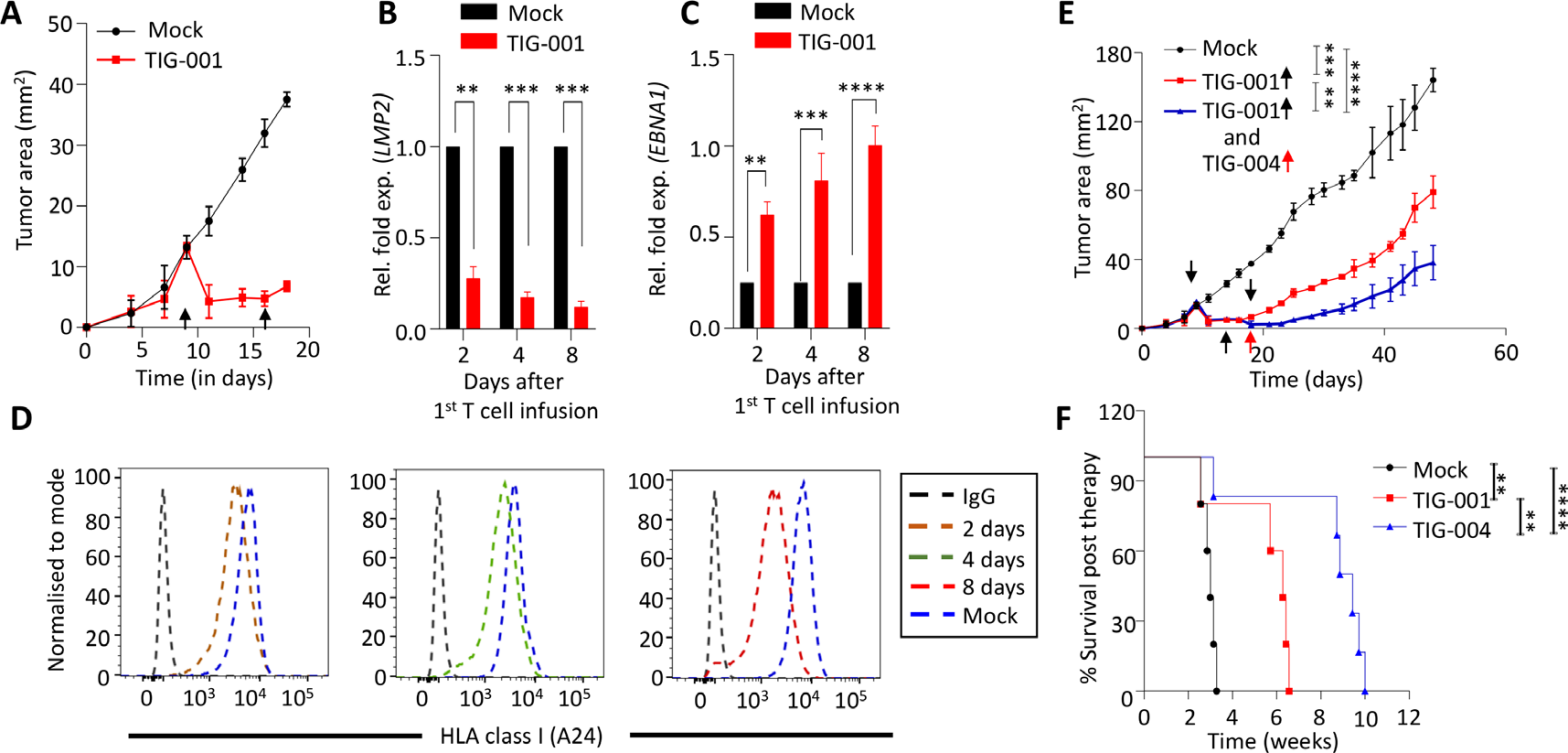
Assessment of ‘switch antigen’ therapy for EBV-associated tumors. (A) Tumor growth kinetics of an SNU719 xenograft following adoptive immunotherapy with EBV-specific T cells (TIG-001). Tumor cells were isolated from the mock (PBS)-treated and TIG-001-treated mice on days 2, 4 and 8 post first T-cell infusion (n=3 mice/ group) and analyzed for transcript expression of EBV genes (*LMP2* (B) and *EBNA1* (C)). Expression of these genes in the T-cell treated samples are represented as the relative fold change compared with their expression in mock-treated samples, wherein housekeeping genes *HPRT1* and *GAPDH* were used as controls. Error bars represent the mean ± SD from three independent experiments. P values were calculated using two-way ANOVA. (D) Surface expression analysis of HLA-A24 was performed using anti-HLA Bw4-specific antibody on tumor cells at the time points described in figure 5A. (E) The impact of switch T-cell therapy on the growth kinetics of SNU719 xenograft were assessed. Tumor-bearing mice were either mock treated or infused with three doses of EBV-specific T cells. These animals were either treated with three consecutive infusions of TIG-001 T cells (the group indicated by the red line, wherein the black arrows indicate the time point of T-cell infusion) or initially treated with two infusions of TIG-001 T cells then switched to TIG-004 T cells for the third infusion (the group indicated by the blue line, wherein the black arrow indicates the time point of infusion of TIG-003 while the red arrow indicates the time point of infusion of TIG-002). Tumor growth is represented as the mean tumor area ± SD from n=9 mice/group. (F) Kaplan-Meier overall survival analysis of mice that were either mock treated, infused with TIG-001 T cells, or infused with a combination of TIG-001 and TIG-004 T cells. The overall survival of these animals (n=6 mice/group) was monitored over the indicated time and statistical significance was analyzed by log‐rank test: **p<0.01; ***p<0.001; ****p<0.0001. PBS was used as mock treatment across all experiments. ANOVA, analysis of variance; EBNA1, EBV-encoded nuclear antigen 1; EBV, Epstein-Barr virus; HLA, human leukocyte antigen; LMP2, latent membrane protein 2.

While observing the effect of EBV-specific T cells on three different models of EBV-associated malignancy, we noticed that tumor outgrowth was significantly reduced after the first T-cell infusion. After the second T-cell infusion, the therapeutic benefit was less pronounced. We hypothesized that this decreasing therapeutic efficacy could be due to the presence of an immunosuppressive tumor microenvironment^37 38^ or HLA and/or EBV antigen loss, which may contribute to immune escape.^39^ To explore this hypothesis, we assessed the expression of EBV genes and HLA class I molecules in tumor cells 2, 4 and 8 days after the first T-cell infusion (figure 5A–D). Using qRT-PCR, we observed a consistent reduction in the expression of LMP2 transcripts, while LMP1 levels were unchanged (figure 5B and online supplemental figure 7A.). Interestingly, we observed a significant increase in EBNA1 transcripts at the same time points (figure 5). We also assessed the expression of HLA-A24 on tumor cells to explore the possibility that adoptive immunotherapy with the HLA-A24-restricted TIG-001 T cells may impose immune pressure on malignant cells. Indeed, data presented in figure 5D and online supplemental figure 6B show consistent downregulation of HLA-A24 following T-cell infusion. However, these tumor cells retained normal HLA-B7 expression (online supplemental figure 7C, D). These data indicate that the EBV tumors, on challenge by allogeneic HLA-matched EBV-specific T cells, can alter viral gene expression alongside downregulation of HLA class I on their cell surface, resulting in immune escape. Based on these observations, we switched the third dose of T-cell therapy from HLA-A24-restricted LMP2-specific TIG-001 T cells to HLA-B7-restricted EBNA1-specific TIG-004 T cells and monitored tumor outgrowth following this ‘switch’ therapy. Data presented in figure 5E clearly demonstrate significant inhibition of tumor outgrowth in mice treated with TIG-004 T cells when compared with the animals treated with multiple infusions of TIG-001 only. Furthermore, mice treated with TIG-004 switch T-cell therapy also showed significantly improved overall survival when compared with animals treated with TIG-001 T cells (figure 5F). Similarly, we observed improved therapeutic efficacy of switch T-cell therapy in the EBV lymphoma model (online supplemental figure 7E, F). Collectively, these data highlight the the superior ability of switch therapy to control tumor progression in vivo, compared with single-product therapy.

### Allogeneic EBV-specific T-cell adoptive immunotherapy effectively inhibits outgrowth of EBV-associated lymphoid malignancies in humanized mice

**Figure 6:**
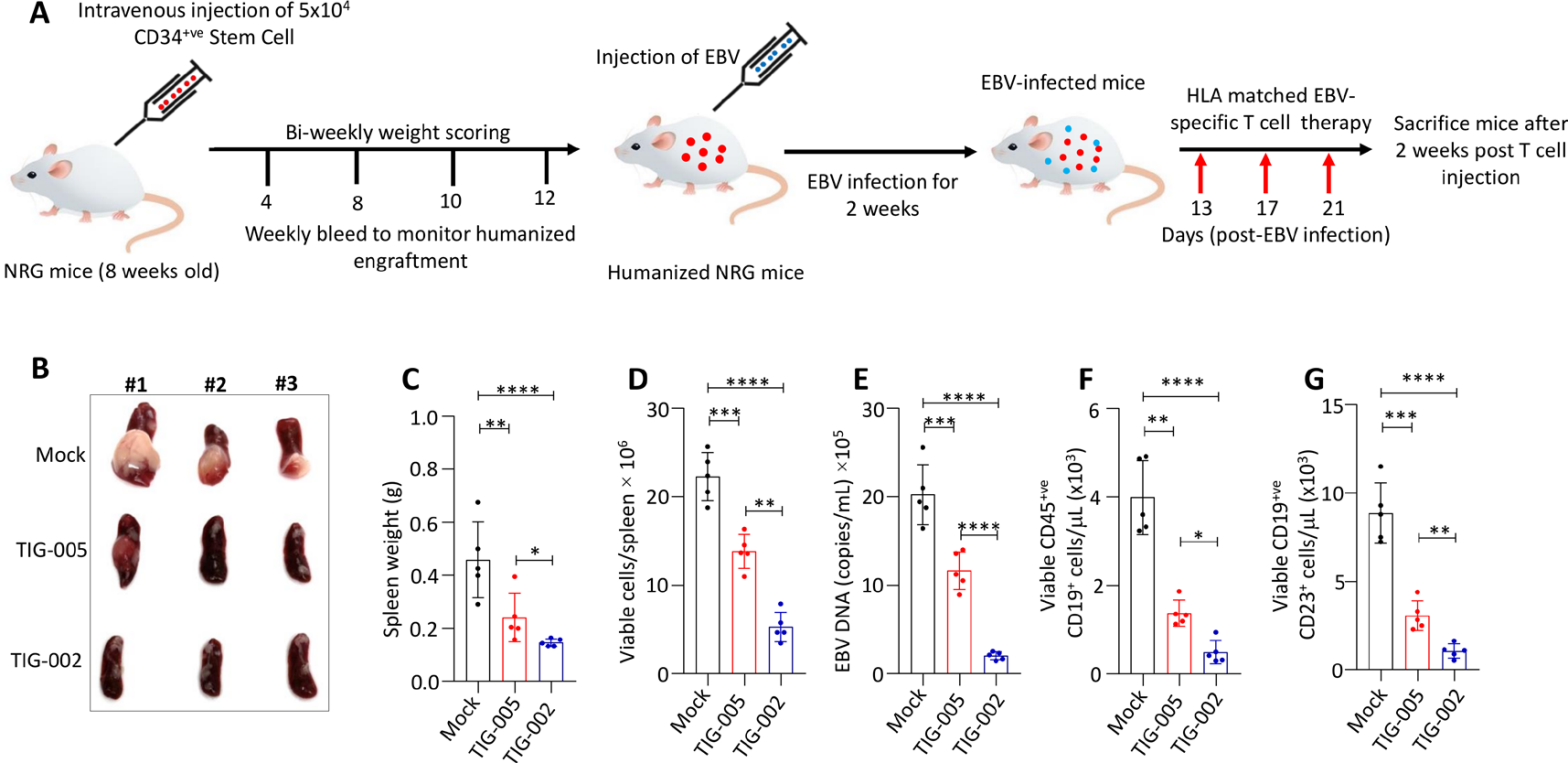
Assessment of therapeutic efficacy of allogeneic EBV-specific T cells in humanized mice-bearing EBV-positive B-cell lymphoma. (A) Schematic showing the reconstitution of the human immune system over 12 weeks in NRG mice using CD34^+^ cells. These mice were intravenously infected with EBV (B95.8 strain), infused with HLA-matched EBV-specific T cells on days 13, 17 and 21 post EBV infection and sacrificed 2 weeks after T-cell therapy. (B) Representative gross morphology of spleens illustrating the size and presence of lymphoid malignancies in the spleen (n=5 mice/group). The first group was mock treated with PBS. Among the treated groups, the second group was infused with three consecutive doses (at intervals of 96 hours) of TIG-005 T cells while the third group initially received two doses of TIG-005 and was later switched to TIG-002 (n=5 mice/group). (C–G) Spleen weight, overall viable cells, EBV load, viable CD45^+^CD19^+^ cells and viable CD19^+^CD23^+^ cells in spleen cells. The statistical significance of tumor weight data was determined using one-way ANOVA: *p<0.05, **p<0.01; ***p<0.001; ****p<0.0001. ANOVA, analysis of variance; EBV, Epstein-Barr virus; NRG, NOD-Rag1^null^ IL2rg^null^.

To further demonstrate the potential therapeutic efficacy of allogeneic EBV-specific T cells, we utilized a humanized mouse model harboring a functionally reconstituted human immune system. The reconstitution of a human immune system was established using intravenous administration of cord blood-derived CD34^+^ stem cells in NRG mice (figure 6A, online supplemental figure 8A). After 12 weeks, these mice were infected with EBV (B95.8 strain) and, following the development of EBV-associated LPD, were adoptively treated with HLA-matched allogeneic EBV-specific T cells (figure 6A). We performed two independent sets of experiments using different donors’ CD34^+^ cord blood cells. In both experiments, NRG mice with LPD were split into three groups (five and six mice in each group in the respective experiments) and were either mock treated or infused with allogeneic T-cell therapy. In the first experiment, among the three groups, the second group of animals was treated with three doses of HLA-A*02:01 and -A*23:01-restricted (TIG-005) allogeneic LMP1-specific and LMP2-specific T cells (2×10^7^ T cells/dose). At the same time, the animals in the third group were given two doses of TIG-005 and a single dose of HLA-A*02:01, HLA-B*40:01 (TIG-002) LMP1-specific, LMP2-specific and EBNA1-specific T cells (2×10^7^ T cells, figure 6B). We observed that the second group of animals, with three doses of the same T cells, showed significantly reduced tumor burden when compared with mock-treated mice (figure 6B–G). Animals in the third group, which were treated with a combination of two different allogeneic EBV-specific T cells, showed significantly reduced tumor burden when compared with both mock-treated and single T-cell product-treated animals (figure 6B–G). These observations were confirmed in a second set of independent experiments in which the second group of animals was treated with three doses of HLA-A*02:01, HLA-B*40:01-restricted (TIG-002) allogeneic LMP1-specific and LMP2-specific T cells (2×10^7^ T cells/dose). At the same time, the animals in the third group were given two doses of TIG-002 and a single dose of HLA-A*11:01, HLA-B*58:01 (TIG-003) LMP1-specific, LMP2-specific and EBNA1-specific T cells (2×10^7^ T cells, online supplemental figure 8B–E). Consistent with our first experiment, we observed that two different allogeneic EBV-specific T-cell products showed significantly improved efficacy in reducing tumor burden when compared with mock-treated and single T-cell product-treated animals. We observed no adverse effects of allogeneic EBV-specific T-cell infusion after multiple infusions in these mice as indicated by their body weight (online supplemental figure 8F, G).

### Blocking the PD-1/PD-L1 axis augments the therapeutic efficacy of allogeneic EBV-specific T cells

**Figure 7:**
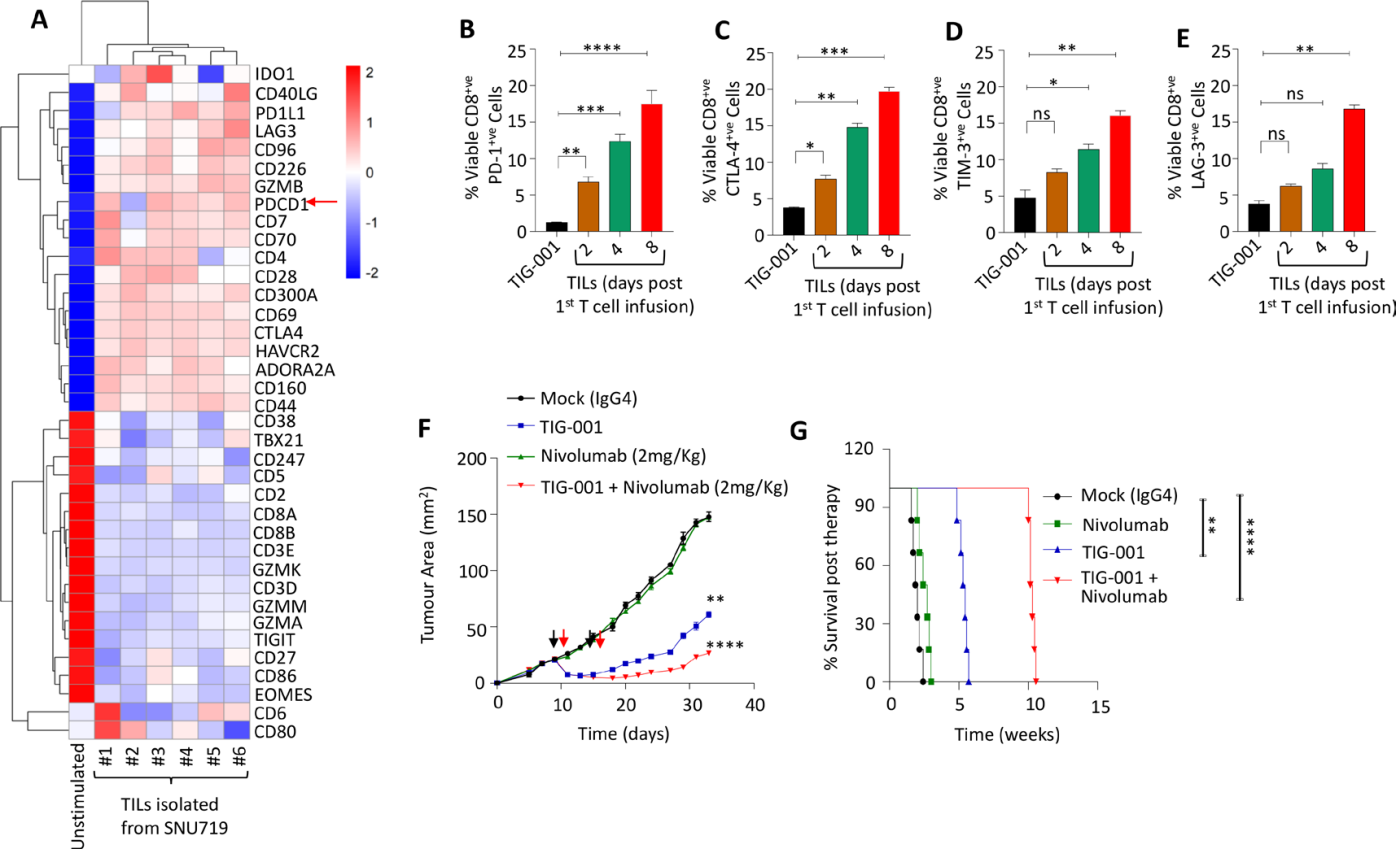
Impact of PD-1 inhibition on therapeutic efficacy of allogeneic EBV-specific T cells in vivo. (A) Heat map representing the gene signature of the checkpoint genes at the transcript level, observed from CD8^+^ TILs, performed using a customized NanoString immune function panel. The TILs were isolated from SNU719-derived tumor xenografts (n=6) 5 days after a single infusion of TIG-001 T cells. The gene expression observed in TILs was compared with T cells in the TIG-001 product. (B–E) The percentage of CD8^+^ TILs expressing PD-1, CTLA4, TIM-3 or LAG3 when compared with TIG-001 T cells. Error bars represent the mean ± SD from three independent experiments. P values were calculated using one-way ANOVA. (F) Tumor growth kinetics of the SNU719 xenograft following monotherapy-based adoptive T-cell therapy, anti-PD-1 (2 mg/kg), or a combination therapy based on two infusions of TIG-001 T cells (2×10^7^ cells/mouse/dose) and two doses of anti-PD-1 (each dose given 24 hours after T-cell treatment). The tumor growth is represented as the mean tumor area ± SD from n=8 mice/group. (G) Kaplan-Meier overall survival analysis of mice bearing the SNU719 xenograft following treatment with monotherapy or combination therapy. PBS was used as mock treatment across all experiments. Survival was monitored over the indicated period (n=5 mice/group) and statistical significance was assessed by log‐rank test: *p<0.05, **p<0.01; ***p<0.001; ****p<0.0001. ANOVA, analysis of variance; CTLA-4, cytotoxic T-lymphocyte-associated protein-4; EBV, Epstein-Barr virus; LAG-3, lymphocyte-activation gene-3; PD-1, programmed cell death protein-1; TILs, tumor-infiltrating lymphocytes; TIM-3, T-cell immunoglobulin and mucin-domain containing-3.

To further assess the interaction of adoptively transferred allogeneic EBV-specific T cells and tumor cells in vivo, we isolated infiltrating T cells from the SNU719 tumors and analyzed their transcriptional signature using NanoString technology. Data presented in figure 7A show a heat map of gene expression in the T-cell therapy products and purified tumor-infiltrating human T cells. This analysis shows that a number of genes and transcription factors associated with effector function were downregulated in tumor-infiltrating human lymphocytes. In contrast, the expression of a number of checkpoint molecules was upregulated in these T cells (figure 7A). We validated the expression of these checkpoint molecules using antibody staining and flow cytometry, and then compared expression levels between the T-cell therapy administered to tumor-bearing mice and tumor-infiltrating lymphocytes isolated at three time points post T-cell therapy infusion. This analysis confirmed the NanoString expression data and showed that tumor-infiltrating human lymphocytes expressed high levels of PD-1, T-cell immunoglobulin and mucin-domain containing-3 (TIM-3), cytotoxic T-lymphocyte-associated protein-4 (CTLA-4) and lymphocyte-activation gene-3 (LAG-3, figure 7B–E).

Based on these observations, we hypothesized that a combination of checkpoint inhibitor and T-cell therapy may offer more therapeutic benefit against EBV-associated tumors. To test our hypothesis, we blocked PD-1 using anti-PD-1 antibody (nivolumab, 2 mg/kg) 24 hours after the administration of the allogeneic EBV-specific T cells and compared tumor growth in these animals to mice that received T cells alone. The combination approach demonstrated significantly reduced (p<0.0001) outgrowth of the GC SNU719 tumors when compared with monotherapy and mock-treated groups (figure 7F). Importantly, we observed that the combination therapy group demonstrated significantly improved overall survival (p<0.0001) when compared with the monotherapy or mock-treated groups (figure 7G). These data highlight that overriding the PD-1/PD-L1 signaling axis can further enhance the therapeutic efficacy of allogeneic EBV-specific T cells.

## Discussion

Virus-specific T-cell therapies have gained importance as a treatment option for refractory viral infections in organ transplant recipients.^28 40 41^ The success of these cellular immunotherapies has been extended to virus-associated hematological malignancies; however, the clinical responses in solid cancer settings are less encouraging.^12 40 42 43^ The manufacturing and clinical use of autologous T-cell therapies are often constrained by multiple factors including the lack of specific immunity due to underlying immunodeficiency and the time required to manufacture these cellular products. This is particularly relevant for patients with virus-associated metastatic solid cancers, who are often severely immunocompromised due to multiple lines of chemo/radiotherapy and are in urgent need of treatment.^11 25 44^ Here we describe a new application of an antigen-presenting platform used to rapidly expand virus-specific T cells. We used this AdE1-LMPpoly platform to expand T cells from healthy blood donors, and demonstrated the potential of these ‘off-the-shelf’ T-cell therapies to treat multiple EBV-associated cancers. We have shown that EBV-specific T cells expanded following stimulation with the AdE1-LMPpoly platform can be successfully used as either monotherapy or in a combination therapy with checkpoint inhibitors for the treatment of EBV-associated cancers of epithelial or lymphoid origin.

Targeting EBNA1, LMP1 and LMP2 is particularly relevant for EBV-associated cancers with a latency II phenotype, which includes NPC GC, HL and NK/T-cell lymphoma.^27 45^ The lack of expression of the immunodominant EBNA3 proteins in latency II malignancies has been recognized as one of the major factors limiting the immune control of these cancers.^46–48^ We have previously shown that T cells directed to EBNA1, LMP1 and LMP2 antigens often display limited polyfunctionality and are highly susceptible to tumor-associated immunosuppressive factors such as galectin-1, which can block proliferation of these effector cells.^49^ Furthermore, we have shown that LMP1 and LMP2-specific CD8^+^ T cells display an impaired capacity to recognize endogenously processed epitopes from EBV-infected cells; however, this can be reversed following stimulation with the AdE1-LMPpoly platform.^49^ These expanded T cells also develop strong resistance against tumor-associated immunosuppressive molecules including galectin-1. Indeed, previous studies with AdE1-LMPpoly-based autologous T-cell therapy in patients with NPC have shown encouraging clinical results, with multiple patients showing disease stabilization.^13^ However, a large proportion of patients with metastatic NPC were unable to receive this therapy due to rapidly progressing disease and severe lymphopenia, which resulted in T-cell therapy manufacturing failure.^12 13^

Based on previous studies of transplant patients in which allogeneic virus-specific T-cell therapies were successfully used for the treatment of multiple virus-associated complications,^41 50–52^ we hypothesized that this strategy could be expanded for the treatment of multiple virus-associated cancers. Our initial in vitro studies showed that EBNA1, LMP1 and/or LMP2-specific T cells expanded from healthy virus carriers efficiently recognized HLA-matched NPC, GC, HL, NK/T-cell lymphoma and EBV-infected LCL. We extended these studies to further demonstrate that these T cells were highly efficient in controlling the outgrowth of multiple EBV-associated cancers including NPC, GC and B cell lymphomas in vivo, in both xenograft models and a humanized murine model. Interestingly, single HLA allele match was sufficient for the immune control of EBV-associated cancers in vivo. However, we noticed that while adoptive T-cell therapy was initially effective, repeat dosing with the same T cells led to immune escape and tumor outgrowth. This immune escape was associated with the loss of EBV antigen expression and downregulation of the HLA class I allele through which the T-cell therapy was restricted. We were able to override this immune escape by switching the allogeneic EBV-specific T-cell therapy to a different HLA restriction and antigen specificity. We successfully demonstrated the therapeutic benefit of switch T-cell therapy in EBV-associated epithelial and lymphoid cancers. It is important to note that switch T-cell therapy has recently been successfully used by Prockop and colleagues for the treatment of rituximab-refractory EBV-associated lymphoma following stem cell transplantation.^26^

Having established the therapeutic potential of allogeneic EBV-specific T cells against multiple latency II malignancies, we next investigated the interactions of adoptively transferred T cells and tumor cells in vivo. These analyses were conducted primarily to study the potential impact of the tumor microenvironment on EBV-specific T cells, which may give us insights into further improving the immune control of malignant cells. We used NanoString technology to assess T-cell-specific gene expression signatures in tumor-infiltrating allogeneic EBV-specific T cells. A GC xenograft model was used for these studies. These studies showed that a number of genes and transcription factors associated with effector T-cell function were downregulated in tumor-infiltrating human lymphocytes. In contrast, the expression of a number of checkpoint molecules was upregulated in these T cells, which provided an important clue for combination therapy. Of particular interest was the upregulation of the *PDCD-1* gene, which was confirmed using cell-surface staining with anti-PD-1 antibody. Based on these observations, we combined allogeneic EBV-specific T-cell therapy and anti-PD-1 therapy and demonstrated significantly improved immune control and long-term survival of tumor-bearing mice.

Data presented in this study provide an important platform for the extension of ‘off-the-shelf’ allogeneic EBV-specific T-cell therapy from transplant settings to virus-associated solid cancers, especially EBV-associated latency II malignancies, which are often difficult to treat in late stages. The allogeneic EBV-specific T-cell therapy described here is specifically targeted against antigens expressed in these malignancies and overcomes many limitations of autologous T-cell therapy. Our therapy features rapid delivery, improved effector functionality and, most importantly, potential use in a combination therapy with checkpoint blockade treatment. In spite of these promising results, one of the potential limitations of these observations, due to a lack of raw material, is that we were unable to directly compare the therapeutic potential of allogenic EBV-specific T cells and non EBV-specific T cells from the same donor in vivo in order to definitively demonstrate that tumor control was mediated by direct recognition of EBV-peptide major histocomptability complex (MHC) complexes by EBV-specific T cells. We are currently in the process of establishing an Australasian T-cell bank for type II EBV-associated malignancies and anticipate that a formal clinical assessment of this off-the-shelf T-cell therapy will initiate within the next 12 months.
