# Supplementary figures and images for: 'Off-the-shelf’ allogeneic antigen-specific adoptive T-cell therapy for the treatment of multiple EBV-associated malignancies
Source: J Immunother Cancer. 2021 Feb 15;9(2):e001608. doi: 10.1136/jitc-2020-001608 (PMC7887372; doi:10.1136/jitc-2020-001608)

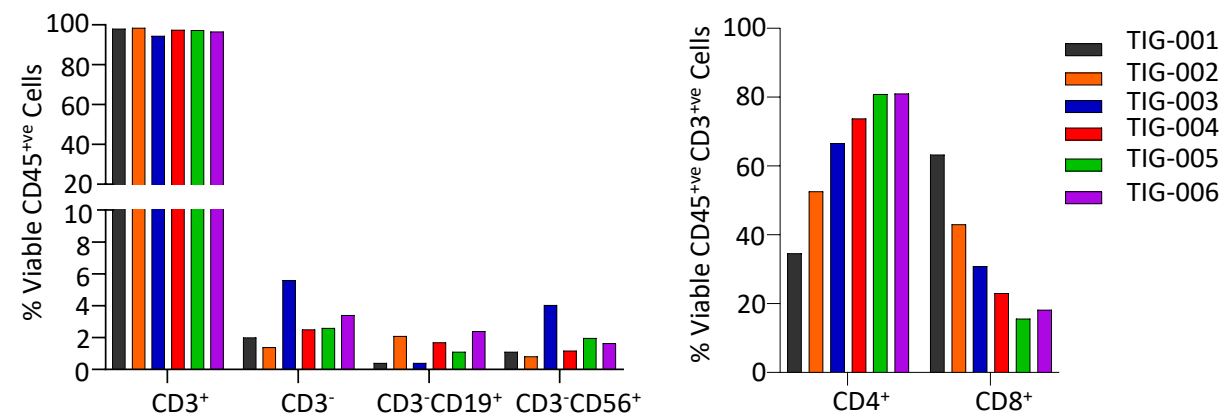

Supp Fig. 1

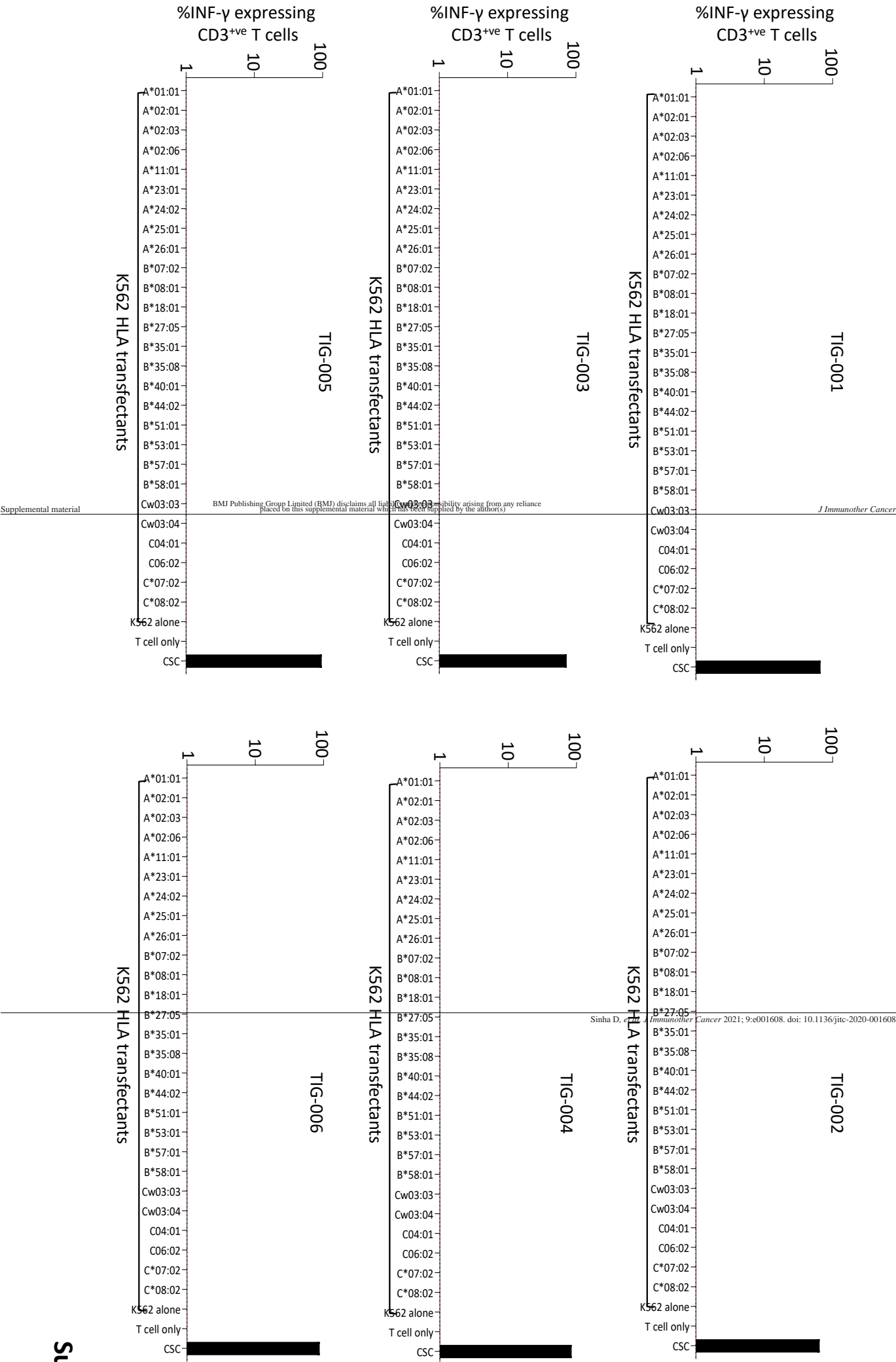

Supp Fig. 2

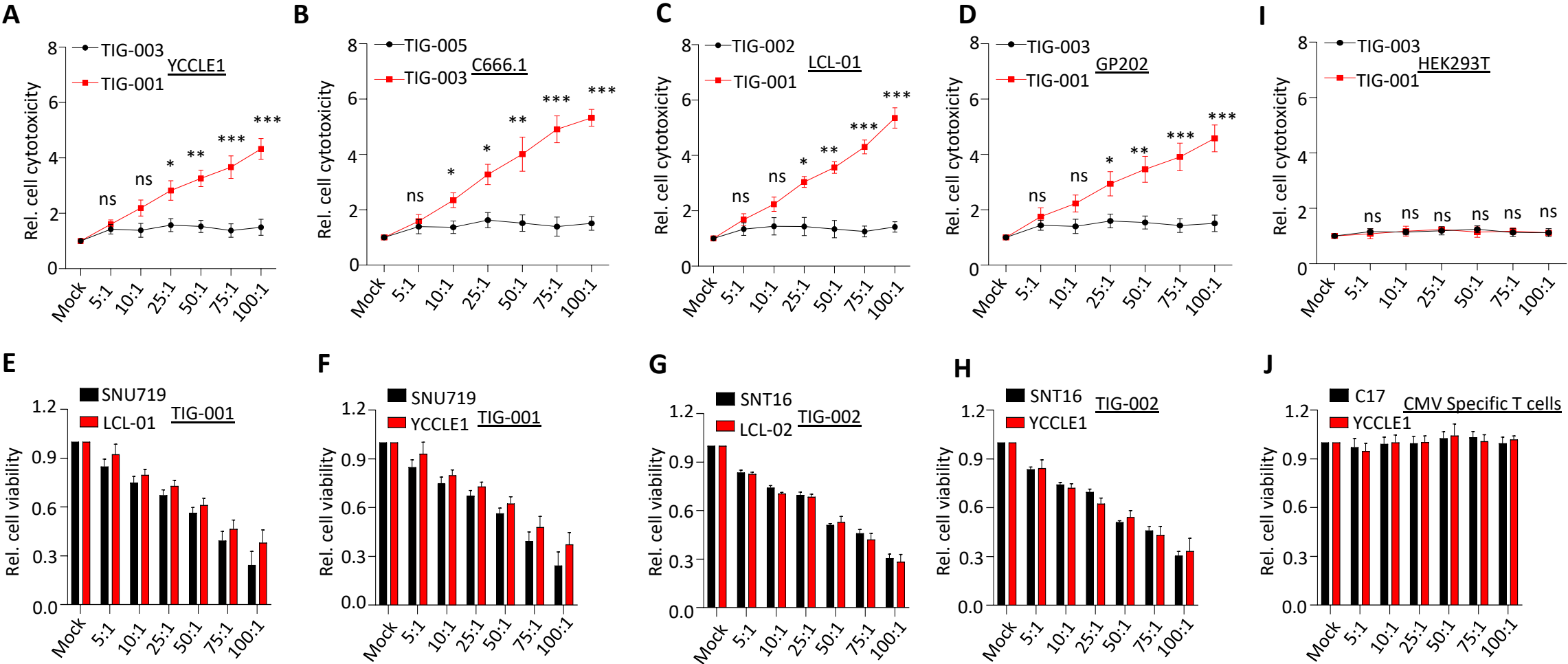

Supp Fig. 3

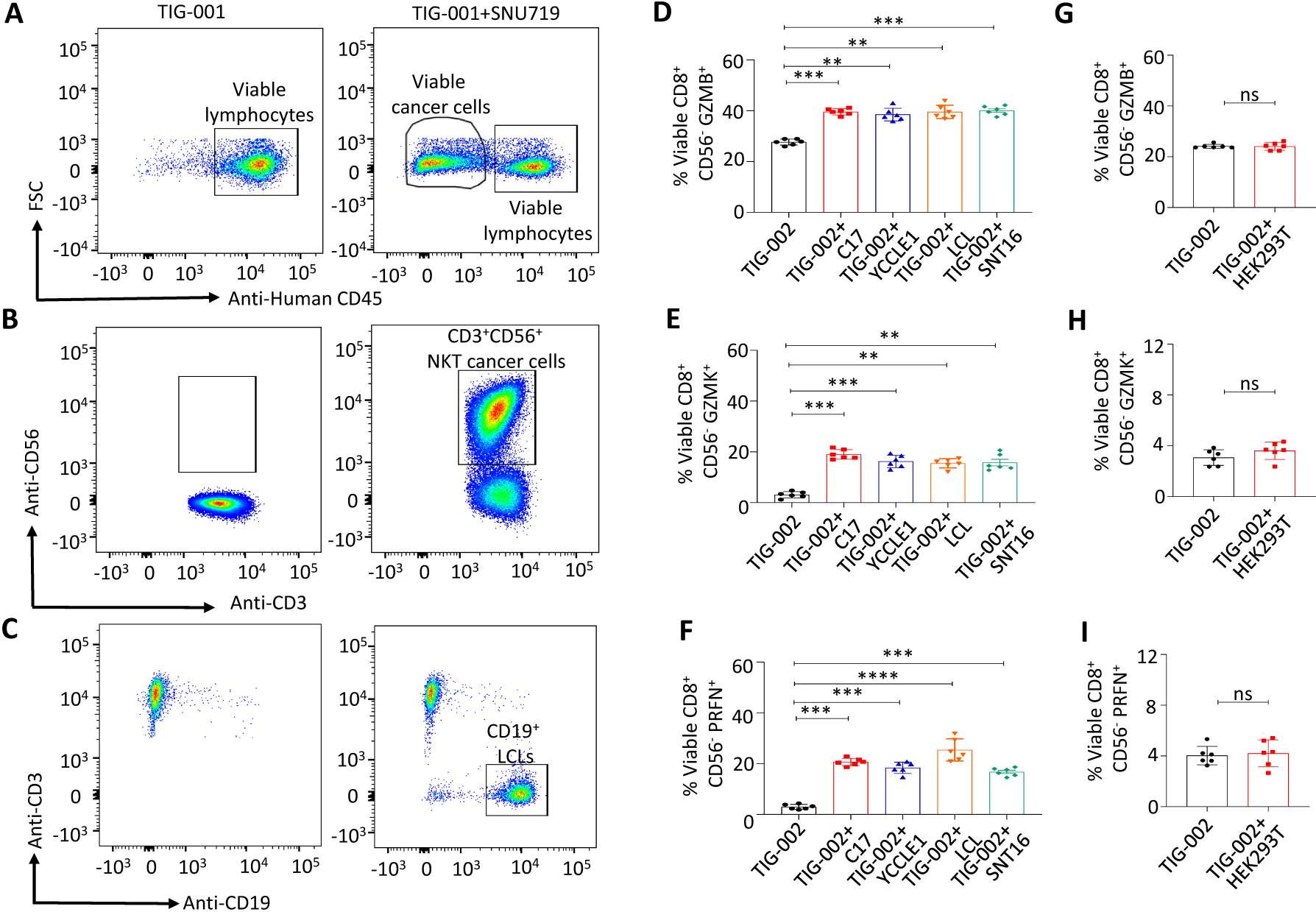

Supp Fig. 4

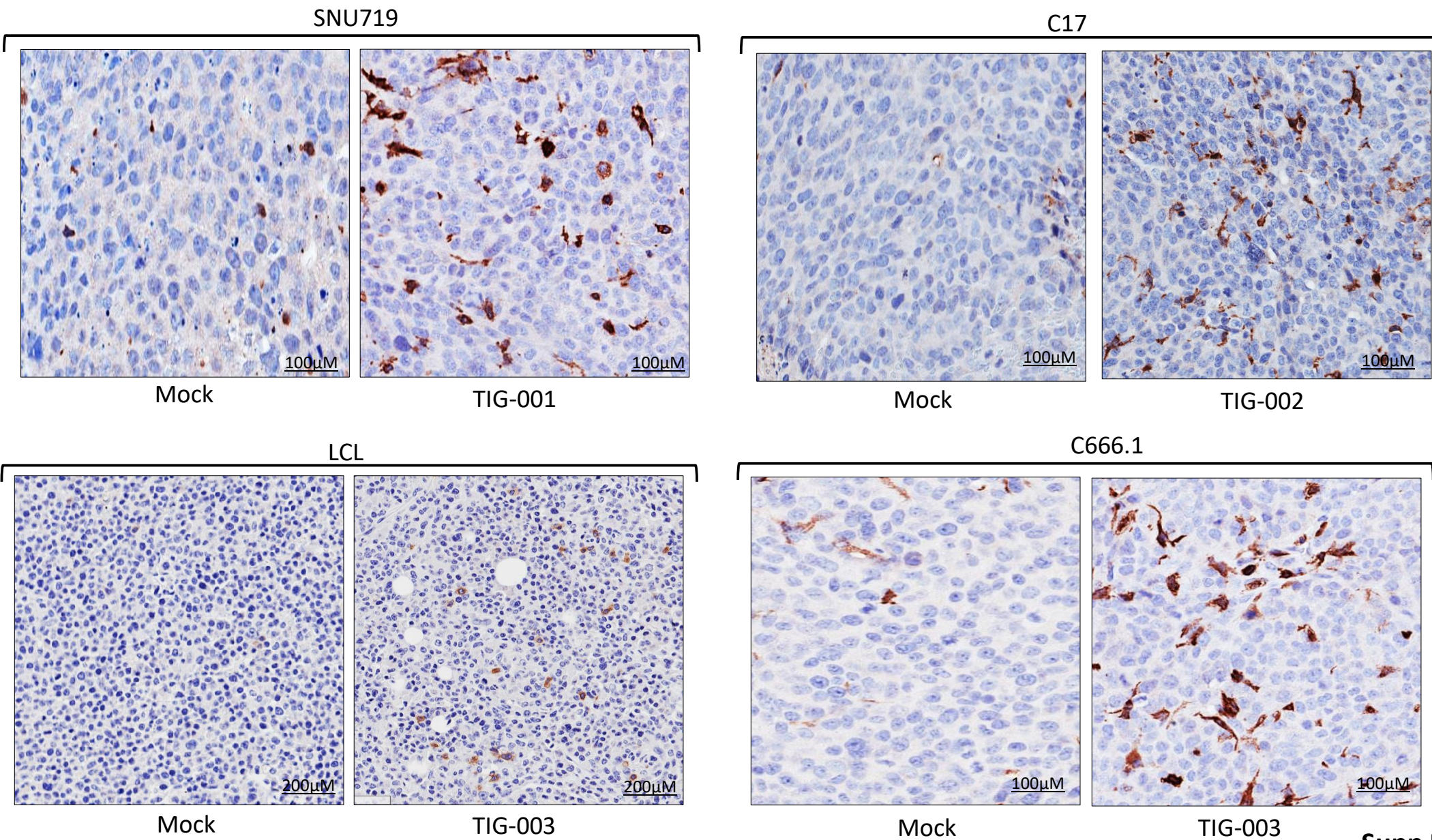

Supp Fig. 5

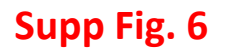

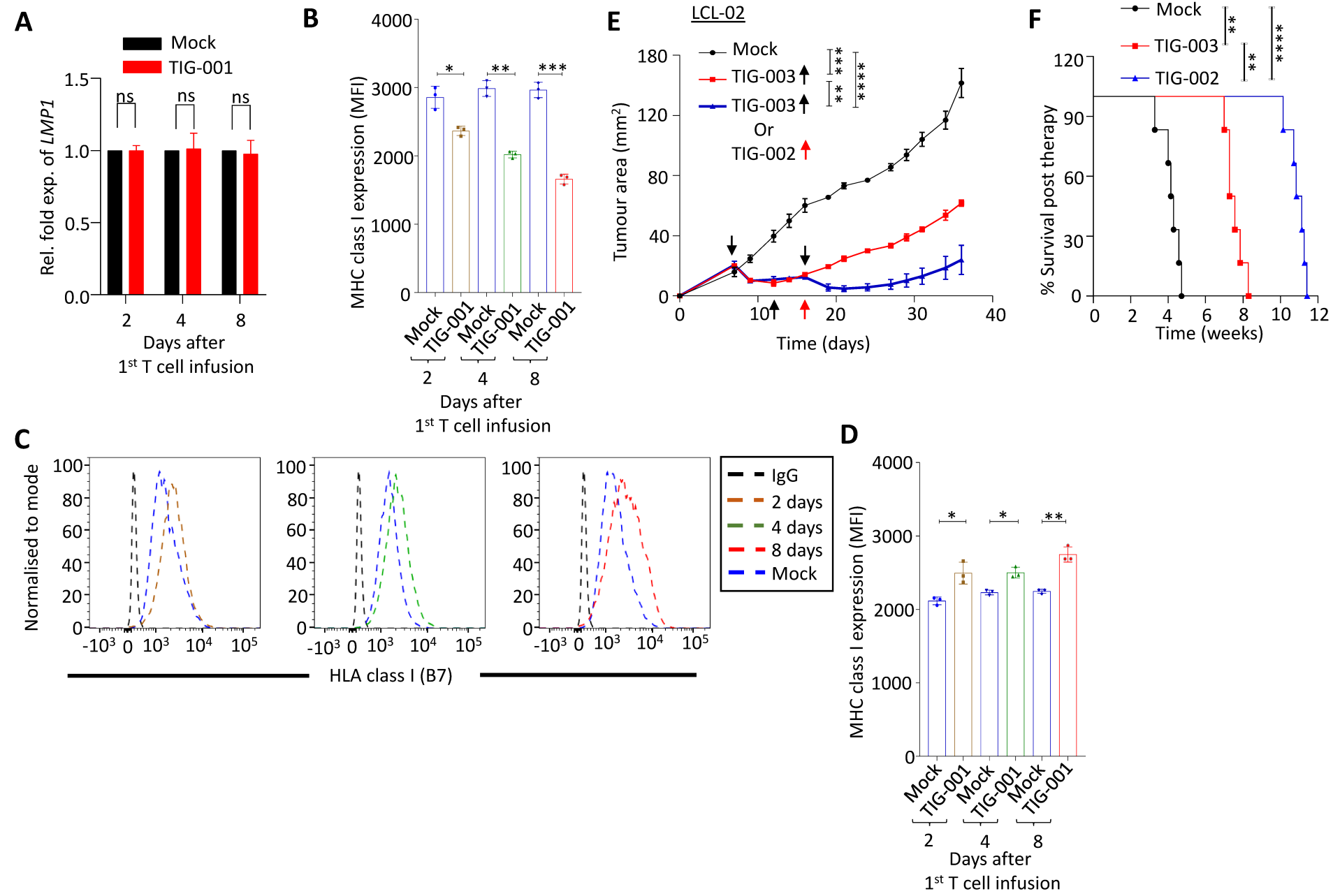

Supp Fig. 7

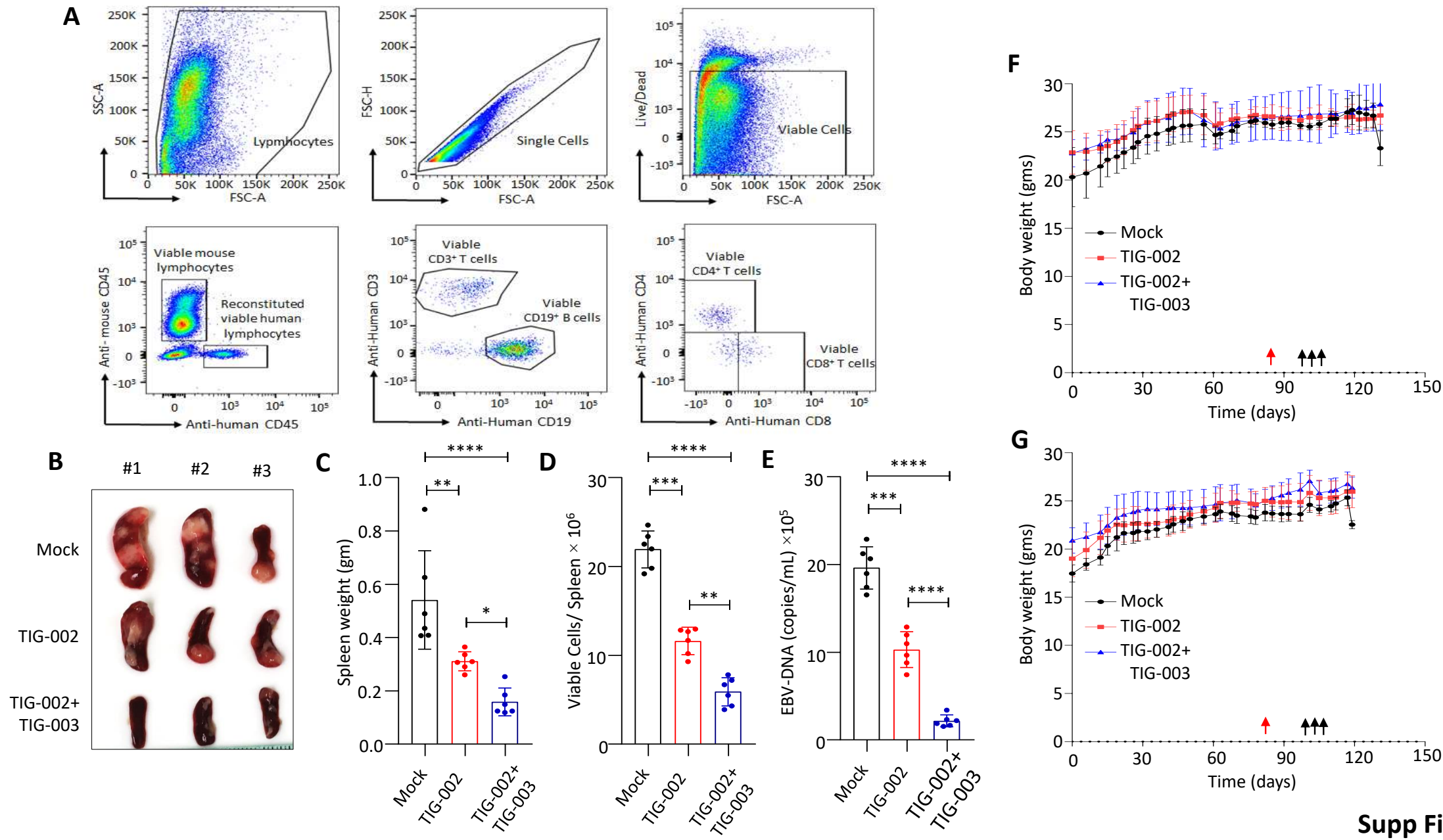

Supp Fig. 8

Supplement: Supplementary data [file jitc-2020-001608supp001.pdf]
