# Supplementary material for: 'Off-the-shelf’ allogeneic antigen-specific adoptive T-cell therapy for the treatment of multiple EBV-associated malignancies
Source: J Immunother Cancer. 2021 Feb 15;9(2):e001608. doi: 10.1136/jitc-2020-001608 (PMC7887372; doi:10.1136/jitc-2020-001608)
Supplement: Supplementary data [file jitc-2020-001608supp002.htm]

***Supplementary
Figure 1: Phenotypic characterization of the “off-the-shelf” EBV-specific T
cells.*** The
phenotypic characteristics of AdE1-LMPpoly-stimulated T cells were assessed
using standard TBNK analysis, measuring the surface expression of CD3 (T
cells), CD8 (CD8+ T cells), CD4 (CD4+ T cells), CD16 and
CD56 (natural killer cells), and CD19 (B cells).

***Supplementary
Figure 2: Assessment of alloreactivity of the “off-the-shelf” EBV-specific T
cells.***Alloreactivity of
AdE1-LMPoly-stimulated EBV-specific T-cell products was assessed using ICS
assay. Each of the T-cell therapy products were incubated with K562 cells
expressing individual HLA class I allele(s) and then assessed for IFN-g using ICS assay. Untransfected K562
cells and T cells alone were used as negative controls, while cell stimulation
cocktail was used as positive control.

***Supplementary
Figure 3: In vitro characterization of the “off-the-shelf” EBV-specific T
cells.***

**(A–B)** The cytotoxicity of HLA-matched
allogeneic EBV-specific T cells was measured by LDH release following their
exposure for 24 hr to EBV-positive cancer cells YCCLE1 (**A**), C666.1 (**B**),
LCL-01 (**C**) and GP202 (**H**) across varying E:T ratios (5:1–100:1). **(E–F)**Cell viability was measured by MTS assay following the exposure of
EBV-positive cancer cells SNU719, LCL-01 (**E**), SNU719 and YCCLE1 (**F**),
SNT16 and LCL-02 (**G**) and SNT16 and YCCLE1 (**H**) for 24 hr to
HLA-matched TIG-001 and TIG-002 allogeneic EBV-specific T cells across varying E:T
ratios (5:1–100:1), as described in **Fig. 2A**. **(I)** The cytotoxicity
of EBV- HEK293T cells was assessed by lactate dehydrogenase release
assay following exposure for 24 hr to HLA-matched and mismatched allogeneic
EBV-specific T cells across varying E:T ratios (5:1–100:1). **(J)** Cell
viability of EBV-negative HEK293T cells assessed by MTS assay following
exposure for 24 hr to HLA-matched and mismatched allogeneic EBV-specific T
cells. PBS was used as mock treatment across all experiments. Error bars
correspond to mean ± SD from three independent experiments. *P-value* were
calculated using Student’s t-test: ns= not significant,\* =p<0.05,
\*\* = p<0.01 and \*\*\* = p<0.001.

***Supplementary
Figure 4: In vitro characterization of the effector function of “off-the-shelf”
EBV-specific T cells.*** **(A–C)** Gating strategies
used to define EBV-positive cancer cells from allogeneic T cells (CD3+)
by flow cytometry: **(A)** Cancer cells of epithelial origin (SNU719,
YCCLE1, C17), which lacked CD45 expression, were defined as CD45–,
compared to CD45+ T cells; **(B)** NK/T cell lymphoma cells
(SNT16) were defined as CD3+CD56+ and; **(C)** LCL
cancer cells were defined as CD19+. **(D–F)** The expression of
effector molecules including granzyme B, granzyme Kand perforin in
allogeneic EBV-specific T cells was assessed following exposure to HLA-matched
EBV-positive cancer cells (E:T of 50:1). *P-values* were calculated using one-way ANOVA **(G–H)** The
expression of effector molecules including granzyme B, granzyme K and perforin
in allogeneic EBV-specific T cells following exposure to EBV-negative HEK293T
cells at an E:T of 50:1. Error bars represent the mean ± SD from three
independent experiments. *P-values* were calculated using Mann-Whitney test: \* = p<0.05, \*\* = p<0.01 and \*\*\* = p<0.001.

***Supplementary
Figure 5: Characterizing the in vivo impact of “off-the-shelf” EBV-specific T
cells.*** Immunohistochemical
analysis was undertaken on the indicated tumour cell line-derived xenograft
cross-section. The presence of CD3+ tumor-infiltrating lymphocytes
(brown stain) in allogeneic T-cell-treated sections is shown, compared to
mock-treated sections. The scales used for magnification are 100 and 200 µM.

***Supplementary
Figure 6: Determining the adverse effects of “off-the-shelf” EBV-specific T
cells in vivo.*** **(A)** The impact of adoptive T-cell therapy
using allogeneic EBV-specific T cells on the overall body weight of the
NOD-SCID mice bearing the xenografts of EBV-positive SNU719 (n=8), C17 (n=9),
C666.1 (n=9) and LCL (n=9). **(B)** The impact of adoptive T-cell therapy
using allogeneic EBV-specific T cells on the organs (liver, spleen and kidney)
of the NOD-SCID mice bearing the xenografts of EBV-positive SNU719 (n=8), C17
(n=9), C666.1 (n=9) and LCL (n=9). Error bars represent the mean ± SD from
three independent experiments*.* *P-values* were calculated using two-way ANOVA. ns= not significant.

***Supplementary
Figure 7: Validating the in vivo efficacy of “switch antigen” therapy using
EBV-specific cytotoxic T cells.*** **(A)** Relative fold expression of *LMP1* observed across the time points
described in **Figure 5A**,in mock- and TIG-001 T cell-treated mice.
Housekeeping genes *HPRT1* and *GAPDH* were used as loading controls.*P-values* were calculated using two-way ANOVA. **(B)** Mean
fluorescence intensity (MFI) of MHC class I (A24) expression observed across
the time points described in **Figures 5A** **and** **5D**.Error
bars represent the mean ± SD from three independent experiments*.* *P-values* were calculated using two-way ANOVA. **(C)** Surface
expression of HLA-B7 was assessed using HLA-Bw6-specific antibody at each time
point. **(D)** MFI of HLA B7 expression observed across different time
points**.** Error bars represent the mean ± SD from three independent
experiments*.* *P-values* were calculated using two-way ANOVA. **(E)** The impact of switch T-cell therapy
on the growth kinetics of an LCL-02-derived xenograft was assessed.
Tumour-bearing mice were either mock treated or infused with three doses of
EBV-specific T cells. These animals were either treated with three consecutive
infusions of TIG-003 T cells (the group indicated by the red line, wherein the
black arrows indicate the time point of T-cell infusion) or initially treated
with two infusions of TIG-003 T cells then switched to TIG-002 T cells for the
third infusion (the group indicated by the blue line, wherein the black arrow
indicates the time point of infusion of TIG-003 while the red arrow indicates
the time point of infusion of TIG-002). Tumour growth is represented as the
mean tumour area ± SD from n≥5 mice/group. **(F)** Kaplan–Meier overall
survival analysis of mice that were either mock treated, infused with TIG-003 T
cells or a combination of TIG-003 and TIG-002 T cells. The overall survival of
these animals was monitored over the indicated time and statistical
significance was analyzed by log‐rank test: \*\* = p<0.01; \*\*\* = p<0.001;
\*\*\*\* = p<0.0001.

***Supplementary
Figure 8: Characterizing the in vivo therapeutic efficacy of “off-the-shelf” T
cells against lymphoid malignancies.*** **(A)** Gating strategies used to phenotype different populations of
human lymphocytes in the peripheral blood of mice following CD34+
cell infusion. **(B)** Representative gross morphology of spleens,
illustrating the size and presence of lymphoid malignancies (n=6 mice/group).
The first group was mock treated with PBS. Among the treated mice, the second
group was infused with three consecutive doses (at intervals of 96 hr) of
TIG-002 T cells while the third group initially received two doses of TIG-002
and later switched to TIG-003. (**C–E)** Spleen weight, number of viable
cells/spleen, and EBV load in spleens. **(F-G)** The impact of adoptive
T-cell therapy using allogeneic EBV-specific T cells on the overall body weight
of humanized mice. *P-values* were calculated using one-way ANOVA: \* = p<0.05, \*\* = p<0.01; \*\*\* =
p<0.001; \*\*\*\* = p<0.0001.

**Supplementary Table 1** HLA restrictions of EBV-specific allogeneic T-cell products
used in the study

|  |  |
| --- | --- |
| **Allogeneic EBV-specific T cell products** | **HLA restrictions of EBV antigen-specific responses** |
| TIG-001 | A\*11:01,24:02;B\*40:01,40:01;C\*03:04,03:04 |
| TIG-002 | A\*02:01, 02:01;B\*40:01,40:01; C\*03:04,03:04 |
| TIG-003 | A\*11:01,33:03;B\*40:01,58:01;C\*03:02,03:04 |
| TIG-004 | A\*03:01,03:01;B\*07:02,07:02;C\*07:01,07:02 |
| TIG-005 | A\*02:01, 23:01;B\*41:02,44:02; C\*05:01,17:01 |
| TIG-006 | A\*02:01, 11:01;B\*13:01,46:01; C\*01:02,03:04 |
